# Transient and Long-Term Risks of Common Physical Activities in People With Low Back Pain

**DOI:** 10.1001/jamanetworkopen.2025.47915

**Published:** 2025-12-09

**Authors:** Pradeep Suri, Andrew K. I. Timmons, Anna M. Korpak, Adrienne D. Tanus, Hannah F. Brubeck, Clinton J. Daniels, Hazel Scott, Daniel Morelli, Nathalia Costa, Paul W. Hodges, Melissa A. Day, Janna L. Friedly, Patrick J. Heagerty, Mark P. Jensen

**Affiliations:** 1 Seattle Epidemiologic Research and Information Center, Veterans Affairs (VA) Puget Sound Health Care System, Seattle, Washington; 2 Rehabilitation Care Services, VA Puget Sound Health Care System, Seattle, Washington; 3 Department of Rehabilitation Medicine, University of Washington, Seattle; 4 Clinical Learning, Evidence, and Research Center, University of Washington, Seattle; 5 Research and Development, VA Puget Sound Health Care System, Seattle, Washington; 6 Clinical Trials Capability Team (ULTRA), University of Queensland, Brisbane, Australia; 7 Centre for Innovation in Pain and Health Research, School of Health and Rehabilitation Sciences, University of Queensland, Brisbane, Australia; 8 Faculty of Health, Medicine and Behavioural Sciences, School of Psychology, University of Queensland, Brisbane, Australia; 9 Department of Biostatistics, University of Washington, Seattle

## Abstract

**Importance:**

Physical activity is considered to have both detrimental and beneficial effects on low back pain (LBP).

**Objectives:**

To examine the association of transient (short-term) risk of 10 common physical activities with participant-reported LBP exacerbations (flares) and to estimate cumulative (long-term) risks of each activity with LBP-related functional limitations at 1-year follow-up.

**Design, Setting, and Participants:**

This prospective, longitudinal case-crossover study nested in a cohort study recruited adults with recent health care visits for LBP at outpatient clinics in Veterans Affairs primary care from March 25, 2021, to September 21, 2023. Participants were followed up for 1 year.

**Exposures:**

The number of hours engaged in each activity in the past 24 hours.

**Main Outcomes and Measures:**

Presence of a participant-reported LBP flare (aim 1) and LBP-related functional limitations at 1-year follow-up (aim 2).

**Results:**

A total of 416 participants (mean [SD] age, 47.5 [10.9] years; 306 [75%] male) completed 9757 surveys during the 1-year follow-up. Each additional hour in which an activity was performed at least once in that hour was associated with greater risk of subsequent flares for lifting more than 10 lb (odds ratio per hour, 1.05; 95% CI, 1.03-1.07), bending, pushing/pulling, twisting, and squatting. Total duration of sitting was associated with lower risk of flares (odds ratio per additional hour sitting, 0.96; 95% CI, 0.94-0.98). However, the mean hours spent in each activity during the initial 8 weeks of the study was not associated with long-term functional limitations at 1-year follow-up.

**Conclusions and Relevance:**

This study found short-term, transient risks of LBP flares associated with some activities but no associations with functional limitations at 1-year follow-up. These findings suggest that people with LBP who engage in more of these activities do not have better or worse long-term outcomes.

## Key Points

**Question:**

Are physical activities associated with transient risks (≤24 hours) of low back pain (LBP) flares and long-term disability?

**Findings:**

This case-crossover study nested within a cohort study of 416 adults with LBP found that, of 10 common activities, greater time spent in lifting, bending, pushing or pulling, twisting, and squatting was significantly associated with greater risks of LBP flares; sitting was significantly associated with lower risk of LBP flares. No activity was significantly associated with LBP-related disability at 1-year follow-up.

**Meaning:**

This study’s results suggest that although some activities were associated with transient risks of a subsequent LBP flare, none were associated with long-term disability.

## Teaser

This case-crossover study nested within a cohort study of adults with low back pain examines and compares the transient and cumulative risks of physical activities on low back pain outcomes.

## Introduction

Low back pain (LBP) is the single greatest contributor to years lived with disability and a common reason for lost work productivity.^1,2,3^ The societal impact of LBP on disability and work roles is attributed not only to its high prevalence^4^ but also to its highly variable course,^5^ which is often characterized by intermittent exacerbations (flares).^3,6,7^

Physical activity is considered to have both detrimental and beneficial effects on LBP. On one hand, a common view among individuals with lived experience of LBP is that activities can trigger the onset or worsening of LBP (flares),^8,9,10^ and some occupational health studies attribute harmful effects to activities such as lifting and bending.^4,11^ On the other hand, randomized clinical trials (RCTs) have found that exercise and activity-based interventions are generally beneficial for LBP prevention and treatment when studying longer-term outcomes up to 1 year after intervention.^12,13,14,15,16^

Different perspectives on whether physical activities are harmful or beneficial for those with LBP might be due to differences in the direction and/or magnitude of the transient (short-term) vs long-term risks of activities on LBP outcomes. For instance, transient and short-lived detrimental effects of physical activity on LBP, such as the occurrence of a flare within 24 hours of activity, may occur alongside long-term beneficial effects of activity on LBP outcomes. This type of association is analogous to the current understanding of the effect of activity on myocardial infarction (MI): vigorous activity is thought to confer a transient increased risk of MI,^17^ alongside a long-term protective effect on MI that occurs with regular exercise.^18^ Most study designs, such as cohort studies and RCTs, target estimation of long-term, cumulative effects of interventions over time frames of months or years and are largely unable to capture transient risks that occur over seconds to hours. However, the short-term and transient risks of physical activities, such as when heavy lifting is performed and LBP begins moments to hours later, may dominate human perception due to the natural tendency for people to infer causation between events that occur in close temporal succession.^19^ Case-crossover designs allow estimation of the transient risks of exposures.^17^

To examine and contrast the association of transient vs cumulative risks of physical activities with LBP outcomes, we conducted a prospective, longitudinal case-crossover study nested within a cohort study. The first study aim examined the association of transient risk of 10 common physical activities with participant-reported exacerbations or LBP flares. The second aim examined the cumulative risks of each activity as performed during the first 8 weeks of the study on LBP-related functional limitations at 1-year follow-up.

## Methods

### Study Sample

We recruited patients of working age (18-65 years) with an index primary care visit for LBP in the Veterans Affairs (VA) Puget Sound Health Care System (VAPSHCS) between March 25, 2021, and September 21, 2023. VAPSHCS serves a 5-state region of the US, but most enrollees reside in Western Washington State. Exclusions were pregnancy, imprisonment, red flag conditions, and severe active comorbidities that would impede the study processes (eMethods in Supplement 1).^20,22^ Collection of data on race and ethnicity was required by the study sponsor; these were participant-reported, using response options that met the sponsor requirements. Participants completed written or oral informed consent. The study was approved by the VAPSHCS and University of Washington institutional review boards. Extended descriptions are provided in the eMethods in Supplement 1.^20^ This report followed the Strengthening the Reporting of Observational Studies in Epidemiology (STROBE) guidelines for cohort studies.^21^

### Study Design

The first study aim used a case-crossover design to estimate associations between transient exposure to 10 common self-reported physical activities and subsequent LBP flares during 1-year follow-up. After a primary care LBP visit, participants completed a baseline survey and scheduled surveys on a randomly generated schedule constrained to a frequency of 3 times per week for weeks 1 to 4, once per week for weeks 5 to 8, and twice per month during months 3 to 12. The time of day when scheduled surveys were sent was randomly varied. When a scheduled survey became available, participants were sent text and/or email messages. As a design feature to minimize selective reporting, each survey was available for only 3 hours, with hourly reminders, after which the survey could no longer be completed. Additionally, participants were able to initiate flare window surveys whenever they experienced a new LBP flare during the 1-year study period. Based on theory and prior work, which assumed effect periods of activities on flares of 2 hours or less^23^ or 24 hours or less,^24,25^ each scheduled and flare window survey inquired about activity exposures in the past 24 hours such that the flare outcome assessment was time-lagged and followed the exposure recall window. If no flare was reported (a control period), surveys inquired about exposures in the 24 hours before the time of survey completion. If a flare was reported (a case period), participants received detailed instructions to orient them to the precise time of flare onset (eMethods in Supplement 1). Participants were asked: “Think about the specific time and day when your current flare of low back pain started.” Participants were then asked to focus on the date and time when their flare began by memory or using aids, such as their smartphone, diary, or calendar, to orient themselves to the precise time of flare onset. Subsequent items then asked about exposures in the 24 hours prior to the participant-reported time of flare onset. Otherwise, the scheduled and flare window surveys included the same content.^20^ This approach calibrated exposure windows to the time of flare onset so that activities performed in response to a flare would not be included in the exposure window. The second study aim used a conventional cohort design to estimate multivariable-adjusted associations between the mean time spent in the 10 activities reported during scheduled surveys during the first 8 weeks of follow-up and functional limitations at 1-year follow-up.

### Outcomes 

The primary outcome for the first aim was participant-reported presence of a flare using a validated flare definition (“A ‘flare’ of low back pain is a worsening of your low back pain that lasts from hours to weeks”).^7^ Participants were then asked to report whether or not a flare was currently present by responding yes or no to the following question: “According to the definition above, are you currently experiencing a flare of your low back pain?” Flare periods identified using this definition are significantly associated with a 2.8-point higher numeric rating scale LBP intensity rating (95% CI, 2.6-2.9) compared with non-flare periods.^7^ The primary outcome for the second aim was LBP-related functional limitations measured by the Roland-Morris Disability Questionnaire (RMDQ) 1 year after the baseline survey.^26,27^

### Physical Activity Exposures

Surveys inquired about 10 activities included in work restriction forms from the federal government,^28^ the 2 most populous states (California^29^ and Texas^30^), and the state in which our hospital system is located (Washington).^31^ Self-report of these activities has been validated in comparison to direct observation.^32,33,34^

Participants were asked to estimate the total hours and minutes spent in each activity during the past 24 hours (eMethods in Supplement 1). The primary activity exposure variable definition differed depending on the activity and was either (1) the number of participant-reported hours in the past 24 hours during which the activity was performed at least once an hour (for lifting ≥10 lb, pushing or pulling, bending, climbing, twisting, squatting, crawling, sitting, standing, and walking) or (2) the number of hours spent in the activity in the past 24 hours (for sitting, standing, and walking). For simplicity of language, we refer to these activity definitions henceforth as the number of hours spent in the activity (eMethods in Supplement 1). Subsequent questions inquired about the total number of times the activity was completed in the past 24 hours.

### Covariates

Covariates included potential confounders based on clinical knowledge and the literature, using core measures recommended by the National Institutes of Health.^26,35^ Baseline covariates included a range of factors related to sociodemographics, LBP history, and mood (eMethods in Supplement 1). Scheduled and flare surveys inquired about time-varying covariates with a 24-hour recall period. To mitigate respondent burden, these surveys used brief, validated 1- or 2-item measures of depressive symptom severity,^36^ posttraumatic stress disorder (PTSD) symptoms,^37^ general stress,^38,39^ fear of movement (kinesiophobia),^40^ catastrophizing,^41^ and self-efficacy.^42^

### Statistical Analysis

We descriptively characterized the study with respect to sociodemographic and clinical factors. Appropriate to the case-crossover design, the first study aim used conditional logistic regression^43^ to estimate associations between activities and flares. As this approach treats each person as their own control, participants without at least 1 case period and 1 control period, although retained in the analysis, are uninformative. Person-level covariates were not included as adjustment variables because they do not change over time. The primary analysis examined activity-flare associations using conditional logistic regression, adjusting for the time-varying covariates of symptoms of depression,^36^ PTSD,^37^ general stress,^38,39^ fear of movement (kinesiophobia),^40^ catastrophizing,^41^ and self-efficacy^42^ in the 24 hours before each assessment. We calculated odds ratios (ORs), 95% CIs, and *P* values for each activity-flare association. These ORs represent the increase in the odds of subsequent flare with each additional hour spent performing that activity in the past 24 hours. Statistical significance of activity-flare associations was determined using the Holm-Bonferroni method to account for multiplicity. Adjusted *P* values were displayed using a threshold of *P* < .05 for significance testing. To examine the robustness of the study findings, we conducted secondary analyses of nonlinear activity-flare associations and the number of times the activity was performed.

The second study aim used linear regression to estimate associations between the mean number of hours with at least 1 occurrence of the activity in the past 24 hours, as reported during scheduled surveys completed during the first 8 weeks of follow-up, and LBP-related functional limitations at 1-year follow-up measured using the RMDQ. Because this was a person-level analysis, analyses adjusted for the baseline RMDQ score and a wide range of other baseline covariates chosen a priori based on conceptual importance, including sociodemographics and factors related to LBP history, mood, and lifestyle (eMethods in Supplement 1).^56,57,58,59,60^ Secondary analyses examined nonlinear associations and repeated the analyses in the subset of participants who were informative in the case-crossover analysis (n = 345).

Missing data were recovered using multiple imputation by chained equations.^44,45^ Primary analyses were of pooled results from imputed datasets. Analyses were performed in R, version 4.5.0 (R Foundation for Statistical Computing). Further details regarding missingness, sample size calculations, and analyses are provided in the eMethods in Supplement 1.

## Results

**Figure 1.**
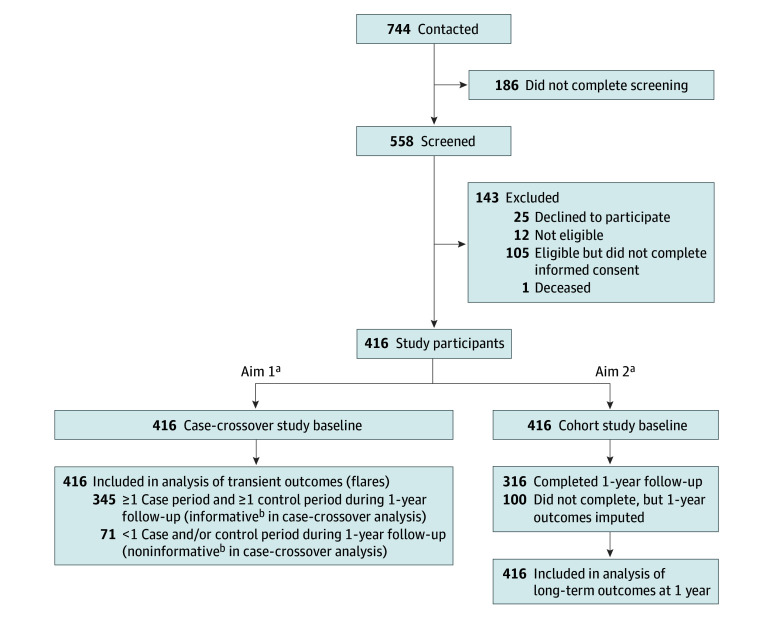
Flow of Study Participants ^a^The branch points showing participants included in aim 1 and aim 2 do not indicate mutually exclusive samples: all 416 participants were eligible to be included in the case-crossover study (aim 1), and all 416 participants were included in the cohort study (aim 2). ^b^In case-crossover analyses using conditional logistic regression, individuals must have had at least 1 case period and 1 control period to be informative; participants without 1 or more case and 1 or more control periods were included in the analysis but are uninformative.

A total of 416 adults (mean [SD] age, 47.5 [10.9] years; 306 [75%] male and 104 [25%] female; 4 [1%] American Indian or Alaska Native, 21 [5%] Asian, 21 [5%] Black or African American, 5 [1%] Native Hawaiian or Other Pacific Islander, 279 [70%] White, and 35 [9%] multiracial) participated in the study) (Table; eTable 1 in Supplement 1). Participants completed 9757 surveys during the 1-year follow-up, including a median (IQR) of 24 (9-30) of 36 possible scheduled surveys (completed within 3 hours of survey delivery) and 2 (1-5) flare window surveys. Of the 416 participants, 345 (83%) had at least 1 case and 1 control period and were informative for the first aim (Figure 1). Participants included in the case-crossover analyses were generally similar to those not included but were more likely to be female (92 [27%] vs 12 [18%]), less likely to report having LBP every day (167 [49%] vs 44 [62%]), and more likely to have lower levels of LBP intensity (mean [SD], 4.3 [2.2] vs 5.1 [2.4] numerical rating scale points) and functional limitations (mean [SD] RMDQ score, 11.8 [5.7] vs 14.1 [6.1]). Three hundred fourteen participants (76%) completed the 1-year RMDQ outcome, but all 416 participants had imputed 1-year outcome data and were included in analyses for the second aim.

**Table.** Characteristics of Study Participants

Characteristic	No. (%) of participants		
	Total (N = 416)	Informative in case-crossover analysis (n = 345)	Not informative in case-crossover analysis (n = 71)
Age, mean (SD), y	47.5 (10.9)	47.5 (10.8)	47.7 (11.5)
Birth sex			
Male	306 (75)	250 (73)	56 (82)
Female	104 (25)	92 (27)	12 (18)
Participant-reported ethnicity of Hispanic or Latino^a^	52 (14)	44 (14)	8 (13)
Race (participant-reported)			
American Indian or Alaska Native	4 (1)	3 (1)	1 (2)
Asian	21 (5)	14 (4)	7 (11)
Black or African American	54 (14)	40 (12)	14 (21)
Native Hawaiian or Other Pacific Islander	5 (1)	4 (1)	1 (2)
White	279 (70)	242 (73)	37 (56)
Multiracial^b^	35 (9)	29 (9)	6 (9)
Marital status			
Married or living with significant other	293 (71)	250 (73)	43 (63)
Never married	37 (9)	32 (9)	5 (7)
Separated, divorced, or widowed	82 (20)	62 (18)	20 (29)
BMI, mean (SD)	30.4 (5.7	30.4 (5.7	30.4 (5.7)
Educational level			
Bachelor’s degree or higher	193 (47)	160 (47)	33 (49)
Less than bachelor’s degree	219 (53)	184 (53)	35 (51)
Employment^c^			
Working now	244 (59)	199 (58)	45 (66)
Retired	93 (23)	77 (22)	16 (24)
Disabled	79 (19)	63 (18)	16 (24)
Other	47 (11)	46 (13)	1 (1)
Income, $			
<75 000	211 (60)	177 (60)	34 (61)
≥75 000	141 (40)	119 (40)	22 (39)
Physical job demands			
Not working	169 (41)	146 (42)	23 (34)
<4	138 (33)	115 (33)	23 (34)
≥4	106 (26)	84 (24)	22 (32)
Job satisfaction			
Not working	169 (41)	146 (42)	23 (34)
Tertile 1 (scores, 5.0-10.0)	102 (25)	85 (25)	17 (25)
Tertile 2 (scores, >10.0-15.0)	70 (17)	59 (17)	11 (16)
Tertile 3 (scores, >15.0-35.0)	72 (17)	55 (16)	17 (25)
Duration of low back pain			
<3 mo	14 (3)	11 (3)	3 (4)
3 mo to <1 y	15 (4)	14 (4)	1 (1)
1 to 5 y	67 (16)	52 (15)	15 (21)
>5 y	318 (77)	267 (78)	51 (73)
Low back pain frequency			
Less than half the days in the past 6 mo	85 (20)	73 (21)	12 (17)
At least half the days in the past 6 mo	119 (29)	104 (30)	15 (21)
Every day or nearly every day in the past 6 mo	211 (51)	167 (49)	44 (62)
RMDQ score, mean (SD)	12.2 (5.8)	11.8 (5.7)	14.1 (6.1)
Low back pain NRS score (mean, 24 h), mean (SD)	4.5 (2.2)	4.3 (2.2)	5.1 (2.4)
Cigarette smoking			
Never smoked	230 (55)	190 (55)	40 (57)
Current smoker	32 (8)	24 (7)	8 (11)
Used to smoke, but have now quit	153 (37)	131 (38)	22 (31)

Abbreviations: BMI, body mass index (calculated as weight in kilograms divided by height in meters squared); NRS, numeric rating scale; RMDQ, Roland Morris Disability Questionnaire.

^a^ Response options provided to participants included Hispanic or Latino, not Hispanic or Latino, unknown, and not reported.

^b^ If participants selected multiple race categories, they were classified as multiracial.

^c^ Not mutually exclusive.

The mean number of flares per year was 8.6. The distribution of flare frequency per year per participant is provided in eFigure 1 in Supplement 1, and the distribution of control periods per year is provided in eFigure 2 in Supplement 1.

**Figure 2.**
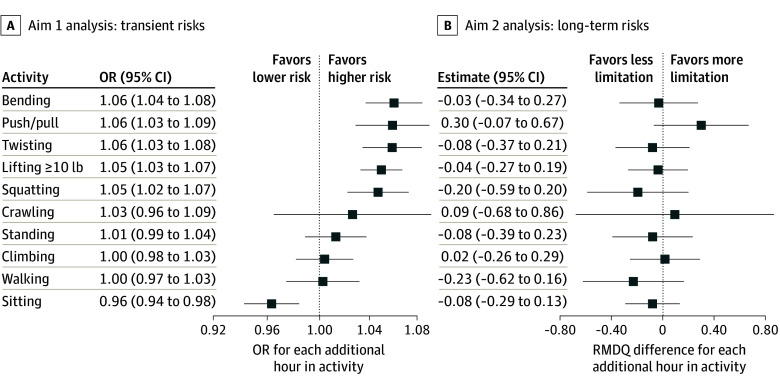
Transient and Long-Term Risks of 10 Common Physical Activities in People With Low Back Pain (LBP) A, Transient risks: associations of hours in activity in the past 24 hours with subsequent flares. The aim 1 analysis is a period-level analysis that uses each person as their own control, accounting for person-level factors by design and adjusting for symptoms of depression,^36^ posttraumatic stress disorder,^37^ general stress,^38,39^ fear of movement (kinesiophobia),^40^ catastrophizing,^41^ and self-efficacy^42^ in the 24 hours before each assessment. The odds ratios (ORs) in the leftmost forest plot are shown on a logarithmic scale, although they may not appear as such due to their close proximity to 1.0. B, Long-term risks: associations of hours in activity at baseline with LBP-related functional limitations at 1-year follow-up. The aim 2 analysis is a person-level analysis that adjusts for baseline age, birth sex, body mass index, cigarette smoking, duration of LBP,^26^ LBP frequency,^26^ the Roland Morris Disability Questionnaire (RMDQ),^27^ depression symptoms,^35,53^ posttraumatic stress disorder symptoms,^54^ kinesiophobia,^55^ catastrophizing,^56^ activity engagement,^57^ pain willingness,^57^ pain-related self-efficacy,^58^ physical demands at work,^59^ and job satisfaction.^60^ Error bars indicate 95% CIs.

eTable 2 in Supplement 1 presents the mean (SD) number of hours in the past 24 hours preceding flare periods and non-flare periods. In adjusted analyses (Figure 2; eTable 2 in Supplement 1), each additional hour in the past 24 hours spent lifting 10 lb or more (OR per hour, 1.05; 95% CI, 1.03-1.07), pushing or pulling (OR, 1.06; 95% CI, 1.03-1.09), bending (OR, 1.06; 95% CI, 1.03-1.08), twisting (OR, 1.06; 95% CI 1.03-1.08), and squatting (OR, 1.05; 95% CI, 1.03-1.08) was significantly associated with greater risk of subsequent participant-reported LBP flares. Conversely, each additional hour spent sitting was significantly associated with lower risk of participant-reported LBP flares (OR, 0.96; 95% CI, 0.94-0.98). For each activity that was significantly associated with flares, the mean (SD) hours spent in that activity in the 24 hours preceding flare periods vs non-flare periods (eg, 2.43 [4.27] hours spent lifting in the 24 hours preceding flare periods vs 1.99 [2.77] hours preceding non-flare periods) were consistent with the directions of association in the adjusted analyses (eTable 2 in Supplement 1). No significant activity-flare associations were found with greater hours spent standing, walking, climbing, or crawling (Figure 2). Unadjusted, complete case, and multivariable analyses were not materially different from the primary analyses (eTables 2-4 in Supplement 1), although multivariable-adjusted activity-flare associations were generally less precise and of smaller magnitude. In secondary analyses examining nonlinear associations (eTable 5 and eFigure 3 in Supplement 1), activities that were significantly associated with flares in the primary analysis generally showed significant dose-dependent associations, with the largest-magnitude risk estimates seen when comparing 1 vs 0 hours spent in the activity, but smaller changes in risk estimates with progressively more hours spent in the activity. In analyses of the number of times each activity was performed, the same activities were identified as being significantly associated with subsequent flares, but the magnitude of associations were generally larger (eTable 6 in Supplement 1).

Participants who completed the 1-year RMDQ outcome for the second study aim were generally similar to those who did not (eTable 7 in Supplement 1). In the primary analyses for this aim, a greater mean number of hours spent in each of the 10 activities studied during the first 8 weeks of the study was not significantly associated with functional limitations at 1-year follow-up after adjusting for baseline functional limitations, other covariates, and multiplicity (Figure 2; eTable 8 in Supplement 1). Notably, none of the activities with detrimental activity-flare associations (lifting, pushing or pulling, bending, twisting, and squatting) or protective associations (sitting) in the case-crossover analysis were significantly associated with 1-year functional limitations. Similar results were found in a complete case analysis (eTable 9 in Supplement 1), in the subset of participants informative in the case-crossover analysis (eTable 10 in Supplement 1), and when examining nonlinear associations (eTable 11 and eFigure 4 in Supplement 1).

## Discussion

This study found transient greater risks of LBP flares in a 24-hour period for greater time spent lifting, pushing or pulling, bending, twisting, and crawling and transient lower risks on LBP flares for a greater time spent sitting. At the same time, greater time spent in these activities during the first 8 weeks of follow-up was not significantly associated with long-term LBP-related functional limitations at 1-year follow-up. These results indicate that although some activities may trigger or prevent LBP flares in the short term, they are not associated with long-term LBP-related functional limitations. The findings provide empirical support for the common view that activities are potential triggers of LBP,^9,10,11^ but they do not support that performing such activities leads to long-term functional limitations, which is consistent with the common public health message that activity generally has beneficial effects on LBP.^46^

The current study’s findings regarding the transient risks of activities are generally consistent with the mixed findings from prior studies. A large, retrospective case-crossover study^23^ found that heavy lifting and awkward postures were associated with the onset of acute LBP. In contrast, a small longitudinal case-crossover study conducted by our team found no significant association between lifting and flares.^25^ Two prior case-crossover studies found no significant associations between standing or walking and flares, as in the current study.^25,47^ On the other hand, the current study’s findings of significant associations between greater time sitting and a lower risk of flares conflict with 2 small case-crossover studies, which found the opposite direction of association.^25,47^ This may be due to differences in the activity exposure periods targeted in these earlier studies: one did not precisely align their activity exposure windows to the time of flare onset, which may have biased activity-flare associations,^25^ and the other would not have reliably captured the 12-hour exposure period immediately prior to flare onset.^47^ The current study’s finding that none of the specific activities investigated were significantly associated with long-term LBP-related functional limitations at 1-year follow-up is also generally consistent with prior studies. Observational studies have found conflicting evidence for the association of workplace activities (eg, lifting) with LBP.^11,48^ RCTs have found small protective effects on LBP outcomes of multimodal interventions that include walking compared with no treatment but detrimental effects when compared with any other treatment^16^ and no effect of interventions to decrease sitting on LBP outcomes.^12^ However, such RCTs are not participant blinded, and their findings may be due to nonspecific effects. Moreover, such RCTs typically combine activity modification with other treatments (eg, education), leaving it unclear which component is responsible for the treatment effect.

No prior study of people with LBP has estimated both the short- and long-term risks of activities in the same sample, leaving open the possibility that differences in prior studies’ conclusions about whether activity is beneficial or detrimental could be due to between-sample differences. Findings from the current study appear to reconcile disparate impressions of how activities affect LBP, illustrating that common patient perceptions regarding the detrimental short-term effects of activities such as lifting on LBP can occur alongside—and be compatible with—the public health message that such activities do not cause long-term problems. Taken together, the current results and past research support that people can engage in physical activities that may be meaningful to them, such as bending down and lifting one’s grandchild, knowing that even if such activities appear to worsen LBP in the short term, they should not cause long-term functional impairments. For some individuals and in certain situations, this leaves choices about activities up to an individual’s personal priorities and preferences. Such choices, however, should also account for knowledge about the broader health benefits of greater activity beyond LBP alone.^49,50^ For instance, our results suggest that sitting may decrease the immediate risk of an LBP flare but has no beneficial association with 1-year long-term outcomes. Nonetheless, personal decisions about sitting should likely also account for the many potential beneficial effects of decreasing sedentary time on other health outcomes, such as cardiovascular disease and mortality.^51^

### Limitations

Although, to our knowledge, this is the largest longitudinal case-crossover study of LBP to date and the only one nested in a cohort study evaluating long-term outcomes, it has limitations. Nearly all pain studies evaluate pain measures using self-report, often using recalled pain during a particular period. Many pain studies also evaluate predictor variables and covariates using self-report measures. Although use of self-report could impart bias in the context of any observational pain study, it is possible that there might be greater potential for such bias when using the case-crossover design, particularly if participants have strong beliefs about activity-LBP associations.^10^ These aspects might partially explain the study findings regarding transient risks of activities but seem less likely to explain the findings of no significant associations with long-term outcomes. Future case-crossover studies of activities in LBP could mitigate potential bias in exposure assessment by using objective monitoring of activities in free-living conditions. Ongoing research on flares by our team will address these potential limitations in future studies by taking measures to blind participants to the study goals and assess sitting, walking, and standing using sensors.^52^ Additionally, although selection bias affecting the current study results is not supported by prior work^22^ demonstrating that the sample was highly representative of the target population of people seen for LBP in VA primary care, it remains possible that the observed associations among activities, flares, and long-term functional outcomes in the current study might not generalize to people in other contexts.

## Conclusions

In this study of people with LBP, we found transient risks on LBP flares for some of the 10 common activities studied but no significant associations between these activities and functional limitations at 1-year follow-up. These findings support that people with LBP can generally engage in these activities, with the knowledge that they are not associated with worse long-term outcomes.

## Notes

Supplement 1. eMethods. Supplementary methods **eTable 1. **Study sample: extended descriptive information **eFigure 1. **Distribution of flare frequency per year per participant **eFigure 2. **Distribution of control periods per year per participant **eTable 2**. Unadjusted and adjusted associations between hours spent in 10 common physical activities during the past 24 hours and subsequent flares, imputed data **eTable 3**. Unadjusted and adjusted associations between hours spent in 10 common physical activities during the past 24 hours and subsequent flares, complete-case analysis **eTable 4.** Multivariable-adjusted associations between exposures and flares of LBP in imputed analysis **eTable 5**. Non-linear associations between hours spent in 10 common physical activities during the past 24 hours and subsequent flares, imputed data **eFigure 3**. Graphical display of non-linear associations between hours spent in 10 common physical activities during the past 24 hours and subsequent flares, imputed data **eTable 6**. Associations between the number of times performing common physical activities during the past 24 hours and subsequent flares, imputed data **eTable 7. **Characteristics of participants completing vs. not completing 1-year RMDQ outcome (Aim 2) **eTable 8.** Adjusted associations between 10 common physical activities performed during the first 8 weeks of study and 1-year functional outcomes as measured by the RMDQ, imputed data **eTable 9.** Adjusted associations between 10 common physical activities performed during the first 8 weeks study and 1-year functional outcomes as measured by the RMDQ, complete case analysis **eTable 10**. Adjusted associations between 10 common physical activities performed during the first 8 weeks of study and 1-year functional outcomes as measured by the RMDQ, among subset of participants informative in the Aim 1 case-crossover analyses, imputed data **eTable 11. **Non-linear associations between 10 common physical activities performed during the first 8 weeks of study and 1-year functional outcomes as measured by the RMDQ, imputed data **eFigure 4**. Graphical display of non-linear associations between 10 common physical activities performed during the first 8 weeks of study and 1-year functional outcomes as measured by the RMDQ, imputed data

Supplement 2.
Group Information

Supplement 3.
Data Sharing Statement
