# Supplementary material for: Transient and Long-Term Risks of Common Physical Activities in People With Low Back Pain
Source: JAMA Netw Open. 2025 Dec 9;8(12):e2547915. doi: 10.1001/jamanetworkopen.2025.47915 (PMC12690428; doi:10.1001/jamanetworkopen.2025.47915)
Supplement: Supplement 1. — eMethods. Supplementary methods eTable 1. Study sample: extended descriptive information eFigure 1. Distribution of flare frequency per year per participant eFigure 2. Distribution of control periods per year per participant eTable 2. Unadjusted and adjusted associations between hours spent in 10 common physical activities during the past 24 hours and subsequent flares, imputed data eTable 3. Unadjusted and adjusted associations between hours spent in 10 common physical activities during the past 24 hours and subsequent flares, complete-case analysis eTable 4. Multivariable-adjusted associations between exposures and flares of LBP in imputed analysis eTable 5. Non-linear associations between hours spent in 10 common physical activities during the past 24 hours and subsequent flares, imputed data eFigure 3. Graphical display of non-linear associations between hours spent in 10 common physical activities during the past 24 hours and subsequent flares, imputed data eTable 6. Associations between the number of times performing common physical activities during the past 24 hours and subsequent flares, imputed data eTable 7. Characteristics of participants completing vs. not completing 1-year RMDQ outcome (Aim 2) eTable 8. Adjusted associations between 10 common physical activities performed during the first 8 weeks of study and 1-year functional outcomes as measured by the RMDQ, imputed data eTable 9. Adjusted associations between 10 common physical activities performed during the first 8 weeks study and 1-year functional outcomes as measured by the RMDQ, complete case analysis eTable 10. Adjusted associations between 10 common physical activities performed during the first 8 weeks of study and 1-year functional outcomes as measured by the RMDQ, among subset of participants informative in the Aim 1 case-crossover analyses, imputed data eTable 11. Non-linear associations between 10 common physical activities performed during the first 8 weeks of study and 1-year functiona [file jamanetwopen-e2547915-s001.pdf]

Supplemental Online Content

Suri P, Timmons AK, Korpak AM, et al. Transient and long-term risks of common physical activities in people with low back pain. *JAMA Netw Open*. 2025;8(12):e2547915.
doi:10.1001/jamanetworkopen.2025.47915

eMethods. Supplementary methods

eTable 1. Study sample: extended descriptive information

eFigure 1. Distribution of flare frequency per year per participant

eFigure 2. Distribution of control periods per year per participant

eTable 2. Unadjusted and adjusted associations between hours spent in 10 common physical activities during the past 24 hours and subsequent flares, imputed data

eTable 3. Unadjusted and adjusted associations between hours spent in 10 common physical activities during the past 24 hours and subsequent flares, complete-case analysis

eTable 4. Multivariable-adjusted associations between exposures and flares of LBP in imputed analysis

eTable 5. Non-linear associations between hours spent in 10 common physical activities during the past 24 hours and subsequent flares, imputed data

eFigure 3. Graphical display of non-linear associations between hours spent in 10 common physical activities during the past 24 hours and subsequent flares, imputed data

eTable 6. Associations between the number of times performing common physical activities during the past 24 hours and subsequent flares, imputed data

eTable 7. Characteristics of participants completing vs. not completing 1-year RMDQ outcome (Aim 2)

eTable 8. Adjusted associations between 10 common physical activities performed during the first 8 weeks of study and 1-year functional outcomes as measured by the RMDQ, imputed data

eTable 9. Adjusted associations between 10 common physical activities performed during the first 8 weeks study and 1-year functional outcomes as measured by the RMDQ, complete case analysis

eTable 10. Adjusted associations between 10 common physical activities performed during the first 8 weeks of study and 1-year functional outcomes as measured by the RMDQ, among subset of participants informative in the Aim 1 case-crossover analyses, imputed data

eTable 11. Non-linear associations between 10 common physical activities performed during the first 8 weeks of study and 1-year functional outcomes as measured by the RMDQ, imputed data

eFigure 4. Graphical display of non-linear associations between 10 common physical activities performed during the first 8 weeks of study and 1-year functional outcomes as measured by the RMDQ, imputed data

This supplemental material has been provided by the authors to give readers additional information about their work.

© 2025 Suri P et al. *JAMA Network Open.*

eMethods. Supplementary methods

The study protocol, including a detailed description of the study methods, has been previously reported.[1] Although the study was observational and not a randomized controlled trial, to promote transparency we registered the trial online at clinicaltrials.gov prior to the start of recruitment (NCT04828330; https://clinicaltrials.gov/study/NCT04828330?term=NCT04828330&rank=1).

Study sample

We recruited participants from among patients of working age (18 to 65 years) with an index primary care visit for LBP in the Veterans Affairs (VA) Puget Sound Health Care System (VAPSHCS). VAPSHCS provides health care to military Veterans in a five-state region of the US (Washington, Alaska, Idaho, Montana, and Oregon), but most enrollees reside in western Washington state. Recruitment was conducted from April 2020 to September 2023. Inclusion criteria included pain in the area of the low back between the lowest ribs and the gluteal fold, with or without radiating leg pain; regular access to a computer, tablet, or smartphone with internet access; having a mobile phone capable of receiving text messages; basic computer literacy; and being able to understand English sufficient to provide informed consent and complete the study assessments. Exclusion criteria were pregnancy; being imprisoned; diagnoses indicating a "red flag" medical condition (spinal malignancy, infection, fracture, or spondyloarthopathy); severe active medical comorbidities or psychiatric illness that would impede completion of frequent electronic surveys over 1-year follow-up; cognitive dysfunction; thoracolumbar spine surgery in the past 1 year; or major orthopedic, abdominal, or chest surgery within the past 6 months. Further details of recruitment are reported elsewhere.[1,2] Using electronic health record data, we have previously shown that study participants were highly comparable to non-participants from the target population with regards to a wide range of sociodemographic and clinical characteristics. [2] Informed consent involved two steps. First, study participants completed informed consent at VAPSHCS. This informed consent was completed either using a hard copy written informed consent document, or remotely using an information statement that the potential participant reviewed and indicated understanding of, with the opportunity to ask questions as needed. Individuals who completed VAPSHCS informed consent were then provided information and a secure code to allow them to access and complete electronic informed consent at the University of Washington (UW), using an information statement that the potential participant reviewed and indicated understanding of, with the opportunity to ask questions if needed. Individuals were considered to have completed informed consent only if they completed consent at both VAPSHCS and UW. The study was approved by the VAPSHCS and University of Washington Institutional Review Boards.

As this study was conducted during the COVID-19 pandemic, recruitment lagged behind that projected. Accordingly, during year 3 of recruitment we applied to the study sponsor (Department of Veterans Affairs, Office of Research and Development, Rehabilitation Research and Development [RR&D]) to expand recruitment to people with LBP from our institution who had not been seen in primary care during the recruitment period (April 2020 to September 2023), including (1) those seen in specialty clinics, and (2) those who had been previously seen in primary care clinics for LBP yet reported ongoing LBP. This subset of study participants with LBP was the study "expanded cohort", distinct from the "original cohort" recruited from primary care as described in the prior paragraph. To remain consistent with our original analytic plan and cohort construction focused on LBP in primary care, the current manuscript reports only results from the "original cohort" of people with LBP seen in primary care clinics, and does not analyze participants from the "expanded cohort". Of note, final recruitment numbers reported in the study registration NCT04828330 include both the original cohort and the expanded cohort, so are larger than the sample size reported in the main article.

Study design

The first study aim used a case-crossover design to estimate associations between 10 common self-reported physical activities and flares of low back pain over 1-year follow-up. This design compared activity exposure status during "case periods" of person-time with activity exposure status during "control periods". Participants were enrolled following their index primary care LBP visit. All subsequent data collection used electronic internet-based surveys. Following a baseline survey, participants completed "scheduled" surveys at a frequency that varied for each of 3 study phases: three times per week for weeks 1-4, once per week for weeks 5-8, and twice per month during months 3-12. The schedule of days

after the baseline visit on which scheduled surveys were sent to participants was randomly generated, constrained by the planned frequency of surveys in each phase. Additionally, the time of day when scheduled surveys were made available to each participant was randomly varied. When a scheduled survey became available for a participant, the participant was sent a text message and/or an email, according to the participant's preference. Each scheduled survey was available for a 3-hour period only, with hourly text and/or email reminders, after which the survey could no longer be completed; this was a design feature used to minimize bias from selective reporting, which was expected to produce substantial missingness of follow-up surveys. In addition to the scheduled surveys, participants were also able to complete participant-initiated *ad hoc* "flare window" surveys whenever they experienced a new LBP flare over the 1-year follow-up period. The survey system did not allow a survey to be completed unless at least 24 hours had elapsed since the participant's last survey was completed. Participants were instructed to complete the flare window surveys within 3 hours of flare onset to reduce recall bias, however, participants were able to complete flare window surveys even after the 3-hour period following flare onset had elapsed. Scheduled and flare window surveys consisted of the same general content.[3]

Case periods were defined as those when a participant reported a flare of LBP either during a scheduled survey or a participant-initiated flare window survey. As flares may last from hours to weeks, and surveys could be completed as frequently as on consecutive days, the same flare could be reported by a participant in two consecutive surveys. Accordingly, only the first reported period in each flare was analyzed as a case period. Control periods included all periods in which a flare was not reported.

An *effect period* is the duration of time within which the effect of an exposure can be expected to manifest as changes in an outcome in a population.[3] As case-crossover studies are focused on transient effects for which timing is critical, exposure windows are selected to closely align with what is known regarding the effect period of an exposure on an outcome. In clinical practice, people with LBP often perceive the effects of activity on LBP as manifesting soon after an activity or on the day after the activity.[4] A ≤24 hour effect period of activities on changes in LBP is supported by two prior studies.[4,5]

Each scheduled and flare window survey inquired about physical activity exposures and covariate exposures in the past 24 hours such that the flare outcome assessment would be time-lagged and always follow the exposure window. If no flare was reported (a control period), surveys inquired about exposures in the past 24 hours prior to the time of survey completion. If a flare was reported in a given survey (a case period), participants were provided information to orient the participant to the precise date and time when the flare began. Participants were asked to "*Think about the specific time and day when your **current** flare of low back pain started*." Participants were then asked to focus on the date and time when their flare began by memory or using aids such as their smartphone, diary, or calendar to orient themselves to the precise time of flare onset. Subsequent question items then asked about exposures in the 24 hours prior to the participant-reported time of flare onset. This approach was taken to ensure precise calibration of exposure windows to the time of flare onset so that activities performed in *response* to a flare would not mistakenly be included in the exposure window; this design feature to mitigate bias was not included in some prior case-crossover studies, including work by our team.[4,5]

The second study aim used a conventional cohort study design to estimate associations between time spent in 10 common self-reported physical activities during the first 8 weeks of follow-up and functional limitations at 1-year follow-up.

Outcomes

The outcome for the first aim was the presence of a flare. Each survey presented a flare definition at the start of the survey, "*A 'flare' of low back pain is a worsening of your low back pain that lasts from hours to weeks*". Participants were then asked participants to report whether or not a flare was currently present, by responding "yes" or "no" to the question "*According to the definition above, are you currently experiencing a flare of your low back pain?* ". The study flare definition has shown strong convergent validity compared to the most common pain domains used in pain research

© 2025 Suri P et al. *JAMA Network Open.*

including LBP intensity , pain interference, and analgesic use.[6] It was adapted from an earlier definition developed by our team through a consensus process incorporating perspectives of experts and people with lived experience of LBP. Participants received information on the flare definition at the time of informed consent, and additional information was available in every survey, if needed, using a pop-up window that provided examples of what would or would not be considered a flare for the purposes of the study.[1] Of note, this flare definition may include either an exacerbation of existing LBP or a new episode of LBP (a return of symptoms after a symptom-free period), consistent with how flares of LBP are most commonly conceptualized.[7]

The primary outcome for the second aim was back-related functional limitations measured by the Roland-Morris Disability questionnaire (RMDQ) at 1 year after the baseline survey. The RMDQ is a validated and widely-used measure in studies of LBP[8] and is recommended by the US National Institutes of Health (NIH) Task Force for Research Standards on Chronic Low Back Pain (TF-CLBP).[9]

Physical Activity Exposures

Scheduled and flare surveys inquired about 10 activities included in work restriction forms from the federal government,[10] the 2 most populous states (California[11] and Texas[12]), and the state where our hospital system is located (Washington).[13] These work restriction forms ask the completing physician or other care provider to make restrictions regarding the maximum number of hours a worker can engage in a task. In order to align with their use in the context of these work restriction forms, we selected the number of hours spent in each activity as the activity exposure variables used in the primary analysis. During study preparation, we solicited input from clinician team members who complete work restriction forms in their normal clinical roles, as well as input from other clinicians with this experience from outside the study team, to understand typical interpretations among clinicians about how to quantify the number of hours spent in activity. Based on this input, the primary exposure variable was defined *a priori* as (1) the number of participant-reported hours in the past 24 hours during which the activity was performed at least once an hour (for lifting ≥10 pounds, pushing/pulling, bending, climbing, twisting, squatting, crawling, sitting, standing, and walking), or (2) the total duration of time (in hours) spent in the activity in the past 24 hours (for sitting, standing, and walking). For the activities of lifting, pushing/pulling, bending, climbing, twisting, squatting, crawling, due to the typically short durations of bouts of these activities and intermittent manner in which these activities are typically done in work contexts, it was thought to be infeasible for participants to mentally sum the total time of many short bouts of these activities. Moreover, based on the clinician input, classifying an hour of these activities (e.g., lifting) as the number of participant-reported hours in the past 24 hours during which the activity was performed at least once an hour was thought to be most consistent with how providers answer these questions about activities in work restriction forms.

Physical activity questions were presented in separate modules in the following order: lifting objects weighing ≥10 pounds, pushing or pulling objects weighing ≥10 pounds, sitting, standing, walking, bending or stooping, climbing, twisting at the waist, squatting or kneeling, and crawling. Self-report of these activities has previously been validated in comparison to direct observation of activities in occupational contexts.[14-16]

For the activity types of lifting ≥10 pounds, pushing/pulling ≥10 pounds, bending/stooping, climbing, twisting, squatting/kneeling, and crawling, each module first asked participants whether they had engaged in the activity at least once in the past 24 hours. Questions specifying weights, such as weight lifted, provided examples of common objects be familiar to residents of the main hospital system catchment area in western Washington, with representative images . For example, common objects weighing about 10 pounds included two reams of standard office copy paper, a medium-sized pumpkin, a large bag of sugar or flour, and a medium-sized bowling ball. If an activity type was reported, subsequent questions inquired about the number of hours in which the activity was done at least one time and the number of times the activity was completed in the past 24 hours. As previously mentioned, the primary exposure variable for each of these activity types was the number of hours in the past 24 hours during which the activity was performed at least once time. For simplicity of language and due to word limits, however, in the manuscript text when we interpret the results of analyses using this activity exposure variable, we refer to the odds ratios of flares associated

© 2025 Suri P et al. *JAMA Network Open.*

with “each additional hour spent” in the activity, rather than the more specific wording of “each additional hour in which the activity was performed at least one time”.

For sitting, standing, and walking, each module asked participants about the total amount of time spent in the activity over the past 24 hours; this was the primary exposure variable for these activity types. For simplicity of language, however, in the manuscript text when we interpret the results of analyses using this activity exposure variable, we refer to the odds ratios of flares associated with “each additional hour spent” in the activity, rather than the more specific wording of “the total duration of time (in hours) spent” in the activity.

Covariates
Covariates included potential confounders based on clinical knowledge and prior research using measures recommended by the NIH TF-CLBP,[9] the HEAL initiative,[17] and/or other validated measures. Covariates included age in years; birth sex; body mass index (BMI) defined as weight in kilograms divided by height in meters squared; smoking status classified as being a current smoker, having quit in the past year, having quit more than 1 year ago, or never having smoked;[9] low back pain duration and frequency;[9] physical demands at work;[18] job satisfaction;[19] LBP intensity measured using the 0 to 10 pain numeric rating scale (NRS);[20] and back-related functional limitations measured using the RMDQ.[8] Baseline assessments of mood included depression symptoms measured by the PROMIS Depression Short Form 8b,[17,21] post-traumatic stress disorder (PTSD) symptoms measured by the PTSD Checklist civilian version,[22] fear of movement measured by the 17-item Tampa Scale of Kinesiophobia (TSK),[23] catastrophizing measured by the 2-item Coping Strategies Questionnaire (CSQ-2),[24] the activity engagement and pain willingness subscales of the Chronic Pain Acceptance Questionnaire 8-item (CPAQ-8),[25] and pain-related self-efficacy using the UW Pain-Related Self-Efficacy Scale 2-item short form (UW-PRSE-2).[26]

Scheduled and flare surveys inquired about time-varying covariates viewed as key potential confounders based on clinical knowledge and prior research, using question items with a 24-hour recall period analogous to the activity questions described above. To mitigate respondent burden given the many detailed surveys completed by participants and their repeated assessments over time, key potential confounders were evaluated used brief, validated 1- or 2-item measures of symptoms of depression,[27] post-traumatic stress disorder (PTSD),[28] general stress,[29,30] fear of movement (kinesiophobia),[31] catastrophizing,[32] and self-efficacy.[33]

<u>*Statistical Analysis*</u>
We calculated descriptive characteristics for the study sample. Appropriate to the case-crossover design, the first study aim used conditional logistic regression[34] to estimate associations between activities and flares. Case-crossover analyses using conditional logistic regression use each person as their own control, so individuals must have at least 1 case period and 1 control period to be informative in the analysis; participants without one case and one control period were included in the analysis but are uninformative. Activity exposure periods were expected *a priori* to be normally distributed for sitting, standing, and walking, and to be right-skewed for other activities. Prior to the primary analyses, we descriptively and graphically evaluated the distribution of time spent in each activity to verify the appropriateness of the analytic approach; this evaluation did indeed show a generally normal distribution for hours spent sitting, standing, and walking, with a right-skewed distribution for other activities (data not shown). The primary activity exposure was the number of hours with at least one occurrence of the activity over the past 24 hours. The outcome was presence versus absence of a flare. In a preliminary step, we examined activity-flare associations using conditional logistic regression models across all survey data obtained over 1-year follow-up, without adjustment for covariates.

Next, we conducted the primary analysis examining activity-flare associations using conditional logistic regression, adjusting for time-varying covariates measured with brief measures selected on conceptual importance, including symptoms of depression,[27] post-traumatic stress disorder (PTSD),[28] general stress,[29,30] fear of movement (kinesiophobia),[31] catastrophizing,[32] and self-efficacy[33] in the 24 hours prior to each assessment**.** We calculated odds ratios (ORs), 95% confidence intervals (95% CIs), and p-values for each activity-flare association. For example, these ORs

© 2025 Suri P et al. *JAMA Network Open.*

reflect the odds of a flare (i.e., having a case [flare] vs. control [non-flare] period) when comparing 0 vs. 1 hours spent doing the activity in the past 24 hours. Similarly, these ORs reflect the odds of a flare (i.e., having a case [flare] vs. control [non-flare] period) when comparing 3 vs. 4 hours spent doing the activity in the past 24 hours. Person-level covariates were not included as adjustment variables, as they do not vary over time. Quantitative variables were analyzed as continuous variables and not categorized. In a preliminary step, we examined activity-flare associations across all survey data obtained over 1-year follow-up, without adjustment for covariates. We based statistical inferences on the multivariable-adjusted analysis for each of the 10 physical activities with flares, using the Holm-Bonferroni method to account for 10 statistical comparisons, with a multiplicity-adjusted p-value threshold of ≤0.05. In order to evaluate for potential biases due to late flare reporting, we conducted a sensitivity analysis limiting participant-initiated *ad hoc* flare window surveys to those reported within 3 hours of flare onset and descriptively evaluated differences in activity-flare point estimates compared to the primary analysis. Secondary analyses to study non-linear associations with flares, examining each activity as a categorical variable defined by cut points based on distribution. We then repeated this approach analyzing the number of times the activity was performed in the past 24 hours.

For the second study aim, we used linear regression to estimate associations between the baseline average number of hours with at least one occurrence of the activity in the past 24 hours during the first 8 weeks of follow-up, as reported during scheduled surveys completed in the first 8 weeks of follow-up, and back-related functional limitations as measured using the RMDQ at 1-year follow-up. The baseline average frequency of each activity was measured over the first 8 weeks of follow-up as it was expected to be more precise than a participant-reported recall of typical activity frequency at a single time point. As this was a person-level analysis, we adjusted for a wide range of factors as potential confounders based on clinical knowledge and prior research, including age, birth sex, BMI, cigarette smoking (current smoker, former smoker, never smoker), duration of low back pain (>5 years vs. ≤5 years),[9] low back pain frequency (less than half the days, at least half the days, every day),[9] baseline RMDQ,[8] depression symptoms measured by the PROMIS Depression Short Form 8b,[17,21] post-traumatic stress disorder (PTSD) symptoms measured by the PTSD Checklist,[22] kinesiophobia measured by the 17-item TSK,[23] catastrophizing measured by the CSQ-2,[24] activity engagement and pain willingness as measured by the CPAQ-8,[25] pain-related self-efficacy using the UW-PRSE-2,[26] physical demands at work (not working vs. <4 vs ≥4),[18] and job satisfaction (grouped by tertiles).[19] Where not otherwise specified in the preceding sentence, quantitative variables were analyzed as continuous variables.

For Aim 1 we examined frequencies of missing data for individual variables and patterns of missing data among all variables that were used as part of the primary, secondary, or sensitivity analyses. Among the variables used in the primary analysis, across the 9150 surveys which were informative in the case-crossover analyses, number of hours sitting was the most commonly missing activity predictor variable (748 missing values [8%]), number of hours crawling was the least commonly missing activity predictor variable (209 missing values [2%]), there was no missingness (0 missing values) for flare status, and the average number missing was 278 (3%). We used multiple imputation with chained equations (MICE) to address missing data, under the assumption that data were missing at random —that is, the probability of missingness could be explained by observed variables in the dataset.[35,36] MICE was chosen because it allows flexible specification of imputation models for different variable types and performs well with complex multivariable data structures.[37] All variables used in primary, secondary, or sensitivity analyses were considered for inclusion in the imputation models, but to improve computational efficiency and model stability variables were only included if they had a correlation with the variable being imputed of 0.05 or greater. Flare was an exception to this rule and was included as a covariate for the imputation model for all variables.[38] All models used the same imputation model method, predictive mean matching using linear mixed models. Several imputation methods were considered, but the method used in the current analysis was chosen for its ability to account for correlated data while effectively and efficiently imputing a variety of variable types.[39]

For Aim 2 a similar approach to missing data was followed. We examined frequencies of missing data for individual variables and patterns of missing data among all variables that were used as part of the primary, secondary, or sensitivity analyses. Among the variables used in the primary analysis, the most commonly missing was RMDQ at 1 year follow-up (n=100, 24%). Variables defined during the first 8-weeks of follow-up surveys had a lower amount of

© 2025 Suri P et al. *JAMA Network Open.*

missingness, with an average of 32 missing values (8%). Baseline survey variables had the lowest amount of missingness, with an average of 3 missing values (1%). MICE was also used for imputation in this aim. All variables used in primary, secondary, or sensitivity analyses were considered for inclusion in the imputation models, but because of a more limited sample size, variables were only included if they had a correlation with the variable being imputed of 0.05 or greater. The RMDQ outcome measure was an exception to this rule and was included as part of the imputation model for all variables. Imputation method for each variable was determined by the form of the variable. Predictive mean matching was used for continuous variables, Bayesian logistic regression was used for binary variables, and Bayesian polytomous regression was used for categorical variables.

For both aims we performed 20 imputations of the missing values, using 20 iterations of the algorithm for each imputation. Trace plots of the mean and standard deviation of the imputed values were examined, and imputed value distributions were examined and compared to non-imputed data to evaluate the imputation. Results using imputed data are pooled from the 20 imputed data sets using Rubin's rules, and all imputations were performed in the R environment using the packages *mice* and *miceadds*.[35,40]

<u>*Sample size and power*</u>

Prior to study execution, sample size was calculated using simulated data with 1000 replications. For the case-crossover analysis (Aim 1), we assumed detectable odds ratios ≥1.5, activity frequencies ≥3%, 80% power, and α=0.005 accounting for 10 statistical comparisons using a Bonferroni correction (1 comparison for each activity), analyzing activity exposure as a binary variable (no exposure to the activity vs. any exposure to the activity). Simulations indicated that 440 participants analyzed would yield >90% power to detect odds ratios of 1.5 for activity frequencies ≥10% and OR=1.8 for activity frequencies ≥3%. For the cohort analysis (Aim 2), simulations assuming 80% power, α=0.005, and clinically meaningful 2.5-point RMDQ differences indicated that n=440 participants analyzed would provide adequate power. These calculations were highly conservative given that our planned analytic approach was to use continuous (instead of binary) activity exposures and the Holm-Bonferroni method to account for multiplicity, such that our actual analyzed sample size (n=416) were expected to be adequately powered.

© 2025 Suri P et al. *JAMA Network Open.*

eTable 1. Study sample: extended descriptive information

	Total (n=416)		Informative in case-crossover analysis[a] (n=345)		Not informative in case-crossover analysis[a] (n=71)	
**Age, mean/SD**						
	47.5	10.9	47.5	10.8	47.7	11.5
**Birth sex**						
Male	306	74%	250	72%	56	79%
Female	104	25%	92	27%	12	17%
Missing	6	1%	3	1%	3	4%
**Ethnicity**						
Hispanic or Latino	52	12%	44	13%	8	11%
Missing	36	9%	26	8%	10	14%
**Race**						
American Indian or Alaska Native	4	1%	3	1%	1	1%
Asian	21	5%	14	4%	7	10%
Black or African-American	54	13%	40	12%	14	20%
Native Hawaiian or Other Pacific Islander	5	1%	4	1%	1	1%
White	279	67%	242	70%	37	52%
Multiracial	35	8%	29	8%	6	8%
Missing	18	4%	13	4%	5	7%
**Marital status**						
Married/Living with significant other	293	70%	250	72%	43	61%
Never married	37	9%	32	9%	5	7%
Separated/Divorced/Widowed	82	20%	62	18%	20	28%
Missing	4	1%	1	0%	3	4%
**Body mass index, mean/SD**						
	30.4	5.7	30.4	5.7	30.4	5.7
**Education**						
High school graduate or GED, or less	21	5%	18	5%	3	4%
Some college, no degree	96	23%	78	23%	18	25%
Occupational/technical/vocational program	37	9%	32	9%	5	7%
Associate degree: academic program	65	16%	56	16%	9	13%
Bachelor's degree	103	25%	84	24%	19	27%
Master's degree or higher	90	22%	76	22%	14	20%
Missing	4	1%	1	0%	3	4%
**Employment[b]**						
Working now	244	59%	199	58%	45	63%
Retired	93	22%	77	22%	16	23%
Disabled	79	19%	63	18%	16	23%
Other	47	11%	46	13%	1	1%
Missing	4	1%	1	0%	3	4%
**Income**						
<$75,000	211	51%	177	51%	34	48%
$75,000+	141	34%	119	34%	22	31%
Missing	64	15%	49	14%	15	21%
**Physical job demands**						
Not working	169	41%	146	42%	23	32%

© 2025 Suri P et al. *JAMA Network Open.*

eTable 1. Study sample: extended descriptive information

	Total (n=416)		Informative in case-crossover analysis[a] (n=345)		Not informative in case-crossover analysis[a] (n=71)	
<4	138	33%	115	33%	23	32%
4+	106	25%	84	24%	22	31%
Missing	3	1%	0	0%	3	4%
**Job satisfaction**						
Not working	169	41%	146	42%	23	32%
Tertile 1: 5.0 to 10.0	102	25%	85	25%	17	24%
Tertile 2: >10.0 to 15.0	70	17%	59	17%	11	15%
Tertile 3: >15.0 to 35.0	72	17%	55	16%	17	24%
Missing	3	1%	0	0%	3	4%
**Duration of low back pain**						
<3 months	14	3%	11	3%	3	4%
3 months to <1 year	15	4%	14	4%	1	1%
1 to 5 years	67	16%	52	15%	15	21%
More than 5 years	318	76%	267	77%	51	72%
Missing	2	0%	1	0%	1	1%
**Low back pain frequency**						
Less than half the days in the past 6 months	85	20%	73	21%	12	17%
At least half the days in the past 6 months	119	29%	104	30%	15	21%
Every day or nearly every day in the past 6 months	211	51%	167	48%	44	62%
Missing	1	0%	1	0%	0	0%
**RMDQ, mean/SD**						
	12.2	5.8	11.8	5.7	14.1	6.1
**Low back pain NRS (Avg. 24hr), mean/SD**						
	4.5	2.2	4.3	2.2	5.1	2.4
**Cigarette smoking**						
Never smoked	230	55%	190	55%	40	56%
Current smoker	32	8%	24	7%	8	11%
Used to smoke, but have now quit	153	37%	131	38%	22	31%
Missing	1	0%	0	0%	1	1%
**Kinesiophobia (TSK), mean/SD**						
	43.3	5.5	43.2	5.3	44.1	6.7
**Catastrophizing (CSQ-2), mean/SD**						
	4.5	3.3	4.5	3.2	4.9	3.6
**Depression (PROMIS-8b), mean/SD**						
	18.4	8.5	18.3	8.5	18.9	8.4
**PTSD (PCL-C), mean/SD**						
	41.0	17.4	40.3	17.0	44.8	18.8
**Activity engagement (CPAQ-8), mean/SD**						
	15.8	5.1	16.0	5.0	14.6	5.6
**Pain willingness (CPAQ-8), mean/SD**						
	12.9	4.9	12.7	4.8	14.3	5.4
**Self-efficacy (UW-PRSE), mean/SD**						
	19.0	5.3	19.2	5.2	18.2	5.6

SD=standard deviation, GED=General education diploma, RMDQ=Roland Morris Disability Questionnaire, NRS=numeric rating scale, hr=hour, TSK=Tampa Scale of Kinesiophobia, CSQ=Catastrophizing Strategies Questionnaire; PROMIS=Patient-Reported Outcomes

© 2025 Suri P et al. *JAMA Network Open.*

eTable 1. Study sample: extended descriptive information

	Total (n=416)	Informative in case-crossover analysis[a] (n=345)	Not informative in case-crossover analysis[a] (n=71)

Measurement Informaiton System, PTSD=Post-traumatic stress disorder, PCL-C=PTSD Checklist Civilian version, CPAQ-8= Chronic Pain Acceptance Questionnaire, 8-item version, UW-PRSE= UW Pain-related Self-Efficacy scale

[a]Participants with both flare and non-flare periods with non-missing data

[b]Not mutually exclusive

© 2025 Suri P et al. *JAMA Network Open.*

eFigure 1. Distribution of flare frequency per year per participant

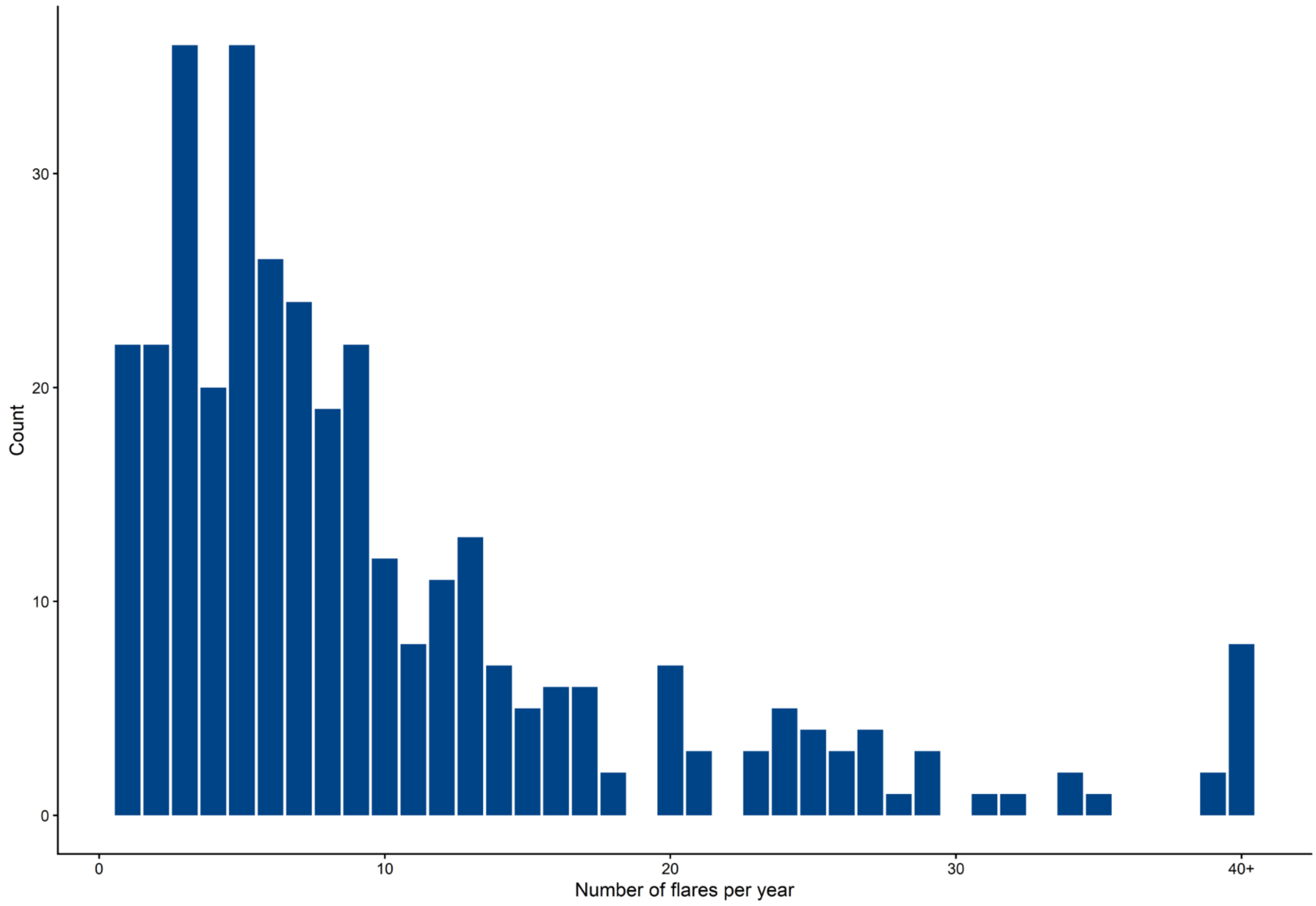

© 2025 Suri P et al. *JAMA Network Open.*

eFigure 2. Distribution of control periods per year per participant

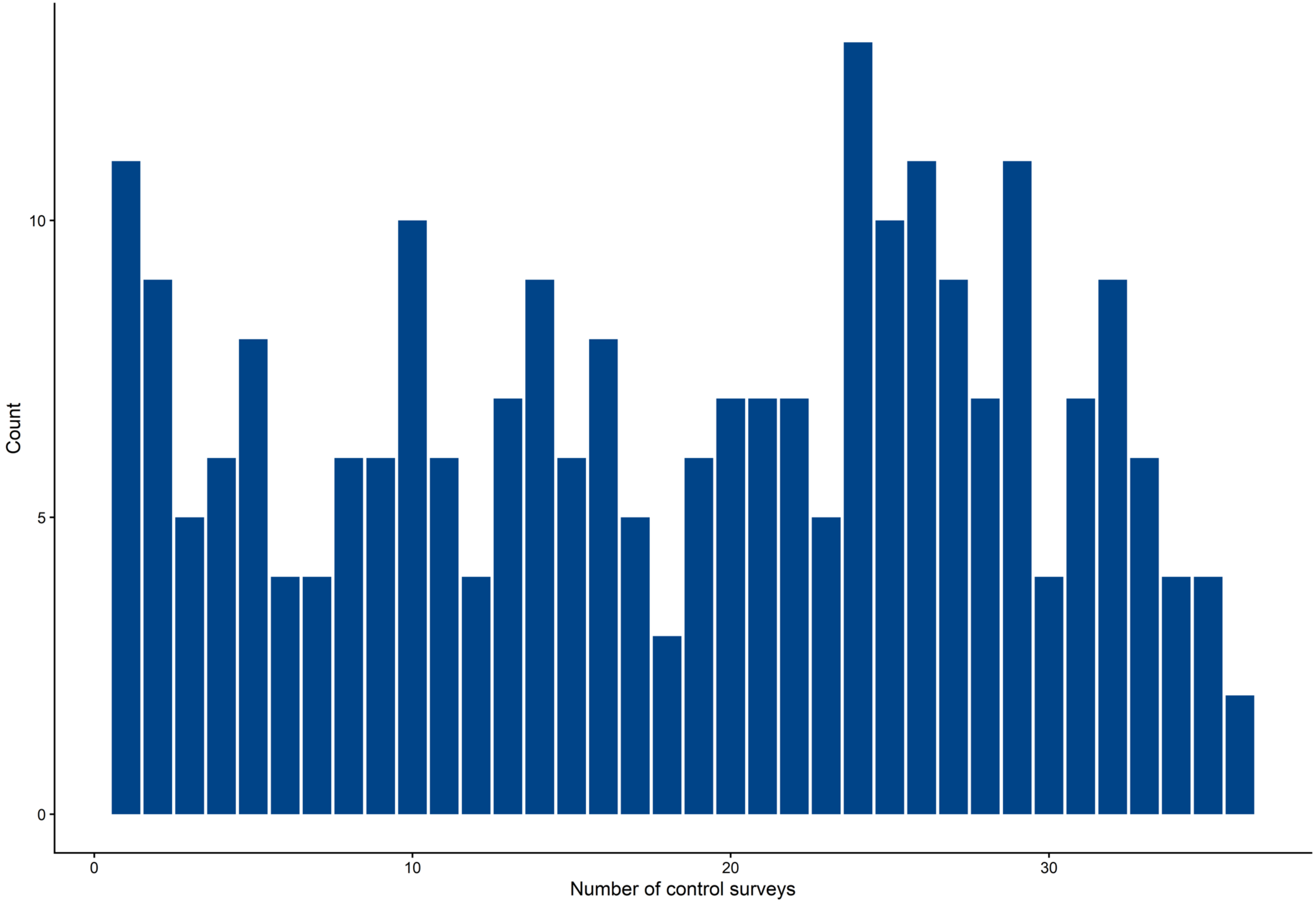

© 2025 Suri P et al. *JAMA Network Open.*

eTable 2. Unadjusted and adjusted associations between hours spent in 10 common physical activities during the past 24 hours and subsequent flares, imputed data[a]

	Total[b]		Non-flare periods[b]		Flare periods[b]		Unadjusted OR (95% CI)		Adjusted OR[c] (95% CI)		p-value	Adjusted p-value[d]
**Climbing**	1.96	(3.30)	1.99	(3.29)	1.88	(3.31)	1.01	(0.98, 1.03)	1.00	(0.98, 1.03)	0.752	1.000
**Twisting**	1.42	(2.92)	1.30	(2.74)	1.70	(3.30)	1.06	(1.04, 1.09)	1.06	(1.03, 1.08)	<0.001	<0.001
**Squatting**	1.29	(2.70)	1.20	(2.49)	1.49	(3.13)	1.05	(1.03, 1.07)	1.05	(1.02, 1.07)	<0.001	<0.001
**Bending**	1.65	(2.88)	1.52	(2.69)	1.95	(3.25)	1.06	(1.04, 1.09)	1.06	(1.04, 1.09)	<0.001	<0.001
**Crawling**	0.23	(1.12)	0.18	(0.90)	0.32	(1.51)	1.04	(0.98, 1.10)	1.03	(0.97, 1.09)	0.389	1.000
**Push/pull**	0.82	(2.11)	0.71	(1.89)	1.09	(2.52)	1.07	(1.04, 1.10)	1.06	(1.03, 1.09)	<0.001	<0.001
**Lifting 10+lbs**	2.12	(3.93)	1.99	(3.77)	2.43	(4.27)	1.05	(1.03, 1.07)	1.05	(1.03, 1.07)	<0.001	<0.001
**Sitting**	6.17	(3.51)	6.39	(3.41)	5.66	(3.68)	0.97	(0.95, 0.99)	0.96	(0.94, 0.98)	<0.001	0.003
**Standing**	3.23	(2.89)	3.25	(2.84)	3.16	(3.01)	1.02	(0.99, 1.04)	1.01	(0.99, 1.04)	0.279	1.000
**Walking**	2.88	(2.46)	2.90	(2.45)	2.82	(2.49)	1.01	(0.98, 1.04)	1.00	(0.98, 1.03)	0.779	1.000

[a]Of 9757 surveys completed by 416 participants, 9150 surveys were completed by 345 participants who were informative in the case-crossover analyses (i.e., the subset of participants who had ≥1 case period and ≥1 control period during 1-year follow-up)

[b]Mean (SD) hours spent in each activity during the past 24 hours (unadjusted). These values are presented for the whole sample of periods, for non-flare periods only, and for flare periods only.

[c]After adjustment for depressive symptom severity,[27] post-traumatic stress disorder (PTSD) symptoms,[28] general stress,[29,30] fear of movement (kinesiophobia),[31] catastrophizing,[32] and self-efficacy.[33]

[d]Holm-Bonferroni method

CI = Confidence interval; OR = odds ratio

© 2025 Suri P et al. *JAMA Network Open.*

eTable 3. Unadjusted and adjusted associations between hours spent in 10 common physical activities during the past 24 hours and subsequent flares, complete-case analysis[a]

	Total[b]		Non-flare periods[b]		Flare periods[b]		Unadjusted OR (95% CI)		Adjusted OR[c] (95% CI)		p-value	Adjusted p-value[d]
**Climbing** (n=8605)[a]	1.95	(3.29)	1.99	(3.29)	1.87	(3.30)	1.01	(0.98, 1.03)	1.00	(0.98, 1.03)	0.702	1.000
**Twisting** (n=8618)[a]	1.41	(2.91)	1.30	(2.74)	1.68	(3.26)	1.06	(1.04, 1.08)	1.06	(1.03, 1.08)	<0.001	<0.001
**Squatting (**n=8634)[a]	1.29	(2.69)	1.21	(2.48)	1.48	(3.12)	1.05	(1.02, 1.07)	1.05	(1.02, 1.08)	<0.001	<0.001
**Bending** (n=8642)[a]	1.65	(2.88)	1.53	(2.70)	1.93	(3.24)	1.06	(1.04, 1.08)	1.06	(1.03, 1.08)	<0.001	<0.001
**Crawling** (n=8645)[a]	0.22	(1.08)	0.19	(0.91)	0.31	(1.41)	1.03	(0.97, 1.10)	1.02	(0.96, 1.09)	0.471	1.000
**Push/pull** (n=8579)[a]	0.81	(2.08)	0.71	(1.88)	1.06	(2.47)	1.07	(1.04, 1.10)	1.06	(1.03, 1.09)	<0.001	0.002
**Lifting 10+lbs** (n=8686)[a]	2.14	(3.95)	2.01	(3.79)	2.43	(4.30)	1.05	(1.03, 1.06)	1.05	(1.03, 1.07)	<0.001	<0.001
**Sitting** (n=8134)[a]	6.07	(3.44)	6.31	(3.34)	5.50	(3.61)	0.97	(0.95, 0.99)	0.96	(0.94, 0.98)	<0.001	0.002
**Standing** (n=8467)[a]	3.17	(2.83)	3.21	(2.79)	3.07	(2.93)	1.02	(0.99, 1.04)	1.01	(0.99, 1.04)	0.328	1.000
**Walking** (n=8449)[a]	2.85	(2.41)	2.89	(2.41)	2.74	(2.42)	1.01	(0.98, 1.04)	1.00	(0.97, 1.03)	0.934	1.000

[a]Surveys with no missing covariate data, completed by 345 participants who were informative in the case-crossover analyses (i.e., the subset of participants who had ≥1 case period and ≥1 control period during 1-year follow-up)

[b]Mean (SD) hours spent in each activity during the past 24 hours (unadjusted). These values are presented for the whole sample of periods, for non-flare periods only, and for flare periods only.

[c]After adjustment for depressive symptom severity,[27] post-traumatic stress disorder (PTSD) symptoms,[28] general stress,[29,30] fear of movement (kinesiophobia),[31] catastrophizing,[32] and self-efficacy.[33]

[d]Holm-Bonferroni method

CI = Confidence interval; OR = odds ratio

© 2025 Suri P et al. *JAMA Network Open.*

eTable 4. Multivariable-adjusted associations between exposures and flares of LBP in imputed analysis (n=9150)

	Total[a]		Non-flare periods[a]		Flare periods[a]		OR[b] (95% CI)		OR[c] (95% CI)		p-value	Adjusted p-value
**Climbing**	1.95	(3.29)	1.99	(3.29)	1.87	(3.29)	1.00	(0.98, 1.03)	0.99	(0.96, 1.01)	0.276	0.903
**Twisting**	1.41	(2.91)	1.29	(2.73)	1.69	(3.29)	1.06	(1.03, 1.08)	1.04	(1.01, 1.07)	0.002	0.022
**Squatting**	1.29	(2.70)	1.20	(2.49)	1.49	(3.12)	1.05	(1.02, 1.07)	1.02	(0.99, 1.05)	0.127	0.636
**Bending**	1.65	(2.87)	1.52	(2.69)	1.95	(3.24)	1.06	(1.04, 1.08)	1.03	(1.01, 1.06)	0.011	0.074
**Crawling**	0.22	(1.11)	0.18	(0.89)	0.32	(1.49)	1.03	(0.96, 1.09)	0.97	(0.90, 1.03)	0.336	0.903
**Push/pull**	0.83	(2.11)	0.71	(1.89)	1.09	(2.53)	1.06	(1.03, 1.09)	1.03	(1.00, 1.06)	0.088	0.529
**Lifting 10+lbs**	2.13	(3.94)	2.00	(3.78)	2.43	(4.28)	1.05	(1.03, 1.07)	1.03	(1.02, 1.05)	<0.001	0.002
**Sitting**	6.17	(3.49)	6.39	(3.40)	5.66	(3.65)	0.96	(0.94, 0.98)	0.97	(0.95, 0.99)	0.002	0.022
**Standing**	3.23	(2.89)	3.25	(2.84)	3.16	(3.00)	1.01	(0.99, 1.04)	1.00	(0.98, 1.03)	0.717	0.903
**Walking**	2.88	(2.46)	2.91	(2.45)	2.80	(2.47)	1.00	(0.97, 1.03)	0.98	(0.95, 1.01)	0.226	0.903

[a]Mean (SD) hours spent in each activity during the past 24 hours (unadjusted). These values are presented for the whole sample of periods, for non-flare periods only, and for flare periods only.

[b]After adjustment for catastrophizing, depression, stress, PCL-2, TSK-2, and PRSE-2

[c]After adjustment for catastrophizing, depression, stress, PCL-2, TSK-2, PRSE-2, and all other activities

CI = Confidence interval; OR = odds ratio

© 2025 Suri P et al. *JAMA Network Open.*

eTable 5. Non-linear associations between hours spent in 10 common physical activities during the past 24 hours and subsequent flares, imputed data[a]

		Total		Non-flare periods		Flare periods		Unadjusted OR (95% CI)		Adjusted OR[c] (95% CI)		p-value	Adjusted p-value
**Climbing hours (24 hours)**	0	3658	(43%)	2459	(41%)	1199	(47%)	1.00	(Ref.)	1.00	(Ref.)	-	-
	1	2497	(29%)	1834	(30%)	663	(26%)	1.15	(0.97, 1.38)	1.19	(0.99, 1.44)	0.063	0.883
	2	556	(6%)	384	(6%)	172	(7%)	1.45	(1.12, 1.88)	1.46	(1.11, 1.93)	0.007	0.111
	3-4	632	(7%)	455	(8%)	177	(7%)	1.24	(0.95, 1.63)	1.23	(0.92, 1.63)	0.165	1.000
	5+	1262	(15%)	896	(15%)	366	(14%)	1.12	(0.89, 1.42)	1.13	(0.88, 1.45)	0.348	1.000
**Twisting hours (24 hours)**	0	4680	(54%)	3378	(56%)	1302	(50%)	1.00	(Ref.)	1.00	(Ref.)	-	-
	1	1949	(23%)	1376	(23%)	573	(22%)	1.80	(1.52, 2.13)	1.81	(1.52, 2.17)	<0.001	<0.001
	2	666	(8%)	443	(7%)	223	(9%)	2.32	(1.83, 2.93)	2.17	(1.69, 2.79)	<0.001	<0.001
	3-4	544	(6%)	345	(6%)	199	(8%)	2.69	(2.07, 3.50)	2.55	(1.93, 3.38)	<0.001	<0.001
	5+	779	(9%)	494	(8%)	285	(11%)	2.44	(1.91, 3.12)	2.36	(1.82, 3.05)	<0.001	<0.001
**Squatting hours (24 hours)**	0	4691	(54%)	3307	(55%)	1384	(54%)	1.00	(Ref.)	1.00	(Ref.)	-	-
	1	2028	(23%)	1462	(24%)	566	(22%)	1.45	(1.23, 1.70)	1.43	(1.21, 1.70)	<0.001	<0.001
	2	661	(8%)	446	(7%)	215	(8%)	1.61	(1.28, 2.02)	1.50	(1.17, 1.91)	0.001	0.020
	3-4	618	(7%)	423	(7%)	195	(8%)	1.65	(1.28, 2.13)	1.66	(1.27, 2.17)	<0.001	0.005
	5+	636	(7%)	411	(7%)	225	(9%)	1.83	(1.41, 2.36)	1.90	(1.44, 2.49)	<0.001	<0.001
**Bending hours (24 hours)**	0	3796	(44%)	2762	(46%)	1034	(40%)	1.00	(Ref.)	1.00	(Ref.)	-	-
	1	2368	(27%)	1685	(28%)	683	(26%)	1.87	(1.59, 2.19)	1.85	(1.56, 2.20)	<0.001	<0.001
	2	796	(9%)	516	(9%)	280	(11%)	2.62	(2.11, 3.26)	2.62	(2.08, 3.30)	<0.001	<0.001

© 2025 Suri P et al. *JAMA Network Open.*

eTable 5. Non-linear associations between hours spent in 10 common physical activities during the past 24 hours and subsequent flares, imputed data[a]

		Total		Non-flare periods		Flare periods		Unadjusted OR (95% CI)		Adjusted OR[c] (95% CI)		p-value	Adjusted p-value
	3-4	795	(9%)	520	(9%)	275	(11%)	2.73	(2.17, 3.43)	2.50	(1.95, 3.20)	<0.001	<0.001
	5+	887	(10%)	569	(9%)	318	(12%)	2.43	(1.94, 3.05)	2.41	(1.90, 3.07)	<0.001	<0.001
**Crawling hours (24 hours)**	0	7774	(90%)	5492	(91%)	2282	(88%)	1.00	(Ref.)	1.00	(Ref.)	-	-
	1	508	(6%)	340	(6%)	168	(6%)	1.51	(1.17, 1.95)	1.51	(1.15, 1.99)	0.003	0.046
	2+	363	(4%)	224	(4%)	139	(5%)	1.37	(0.97, 1.94)	1.19	(0.82, 1.75)	0.361	1.000
**Push/pull hours (24 hours)**	0	5762	(67%)	4178	(69%)	1584	(62%)	1.00	(Ref.)	1.00	(Ref.)	-	-
	1	1616	(19%)	1090	(18%)	526	(21%)	1.47	(1.26, 1.72)	1.54	(1.31, 1.82)	<0.001	<0.001
	2	491	(6%)	323	(5%)	168	(7%)	1.61	(1.26, 2.07)	1.63	(1.25, 2.12)	<0.001	0.007
	3-4	346	(4%)	220	(4%)	126	(5%)	1.64	(1.22, 2.19)	1.62	(1.18, 2.23)	0.003	0.046
	5+	364	(4%)	208	(3%)	156	(6%)	2.13	(1.59, 2.86)	2.00	(1.46, 2.74)	<0.001	<0.001
**Lifting 10+lbs hours (24 hours)**	0	3885	(45%)	2839	(47%)	1046	(40%)	1.00	(Ref.)	1.00	(Ref.)	-	-
	1	1903	(22%)	1318	(22%)	585	(22%)	1.61	(1.37, 1.88)	1.73	(1.46, 2.05)	<0.001	<0.001
	2	830	(10%)	555	(9%)	275	(11%)	2.01	(1.64, 2.46)	1.98	(1.59, 2.46)	<0.001	<0.001
	3-4	850	(10%)	551	(9%)	299	(11%)	2.03	(1.65, 2.49)	2.09	(1.68, 2.60)	<0.001	<0.001
	5+	1218	(14%)	808	(13%)	410	(16%)	2.14	(1.76, 2.61)	2.16	(1.75, 2.66)	<0.001	<0.001
**Sitting hours (24 hours)**	0-1	498	(6%)	233	(4%)	265	(11%)	1.00	(Ref.)	1.00	(Ref.)	-	-
	2-3	1251	(15%)	808	(14%)	443	(18%)	0.61	(0.47, 0.80)	0.59	(0.44, 0.78)	<0.001	0.005

© 2025 Suri P et al. *JAMA Network Open.*

eTable 5. Non-linear associations between hours spent in 10 common physical activities during the past 24 hours and subsequent flares, imputed data[a]

		Total		Non-flare periods		Flare periods		Unadjusted OR (95% CI)		Adjusted OR[c] (95% CI)		p-value	Adjusted p-value
	4-5	1996	(25%)	1383	(24%)	613	(25%)	0.55	(0.42, 0.71)	0.50	(0.38, 0.66)	<0.001	<0.001
	6-7	1908	(23%)	1437	(25%)	471	(20%)	0.44	(0.33, 0.58)	0.40	(0.30, 0.54)	<0.001	<0.001
	8-9	1296	(16%)	971	(17%)	325	(13%)	0.52	(0.39, 0.70)	0.47	(0.34, 0.64)	<0.001	<0.001
	10+	1185	(15%)	889	(16%)	296	(12%)	0.50	(0.37, 0.68)	0.44	(0.32, 0.61)	<0.001	<0.001
**Standing hours (24 hours)**	0	674	(8%)	414	(7%)	260	(10%)	1.00	(Ref.)	1.00	(Ref.)	-	-
	1	1938	(23%)	1354	(23%)	584	(23%)	0.88	(0.69, 1.12)	0.84	(0.65, 1.08)	0.173	1.000
	2	1517	(18%)	1062	(18%)	455	(18%)	1.12	(0.86, 1.45)	1.01	(0.77, 1.34)	0.922	1.000
	3	1228	(15%)	889	(15%)	339	(14%)	1.10	(0.84, 1.45)	1.07	(0.80, 1.44)	0.636	1.000
	4	990	(12%)	730	(12%)	260	(10%)	1.00	(0.75, 1.34)	0.87	(0.64, 1.20)	0.399	1.000
	5+	2120	(25%)	1515	(25%)	605	(24%)	1.10	(0.84, 1.44)	1.05	(0.78, 1.40)	0.755	1.000
**Walking hours (24 hours)**	0	506	(6%)	326	(5%)	180	(7%)	1.00	(Ref.)	1.00	(Ref.)	-	-
	1	2034	(24%)	1405	(24%)	629	(25%)	1.27	(0.97, 1.66)	1.24	(0.92, 1.65)	0.152	1.000
	2	1906	(23%)	1324	(22%)	582	(23%)	1.36	(1.02, 1.81)	1.32	(0.98, 1.80)	0.071	0.926
	3	1369	(16%)	988	(17%)	381	(15%)	1.26	(0.93, 1.70)	1.27	(0.92, 1.76)	0.143	1.000
	4	983	(12%)	714	(12%)	269	(11%)	1.29	(0.94, 1.77)	1.32	(0.94, 1.86)	0.104	1.000
	5+	1651	(20%)	1193	(20%)	458	(18%)	1.28	(0.94, 1.74)	1.20	(0.86, 1.67)	0.275	1.000

[a]Of 9757 surveys completed by 416 participants, 9150 surveys were completed by the 345 participants who were informative in the case-crossover analyses (i.e., the subset of participants who had ≥1 case period and ≥1 control period during 1-year follow-up)

© 2025 Suri P et al. *JAMA Network Open.*

eTable 5. Non-linear associations between hours spent in 10 common physical activities during the past 24 hours and subsequent flares, imputed data[a]

	Total	Non-flare periods	Flare periods	Unadjusted OR (95% CI)	Adjusted OR[c] (95% CI)	p-value	Adjusted p-value

[b]Mean (SD) hours spent in each activity during the past 24 hours (unadjusted). These values are presented for the whole sample of periods, for non-flare periods only, and for flare periods only.

[c]After adjustment for symptoms of depression,[27] post-traumatic stress disorder (PTSD),[28] general stress,[29,30] fear of movement (kinesiophobia),[31] catastrophizing,[32] and self-efficacy.[33]

CI = Confidence interval; OR = odds ratio

© 2025 Suri P et al. *JAMA Network Open.*

eFigure 3. Graphical display of non-linear associations between hours spent in 10 common physical activities during the past 24 hours and subsequent flares, imputed data

© 2025 Suri P et al. *JAMA Network Open.*

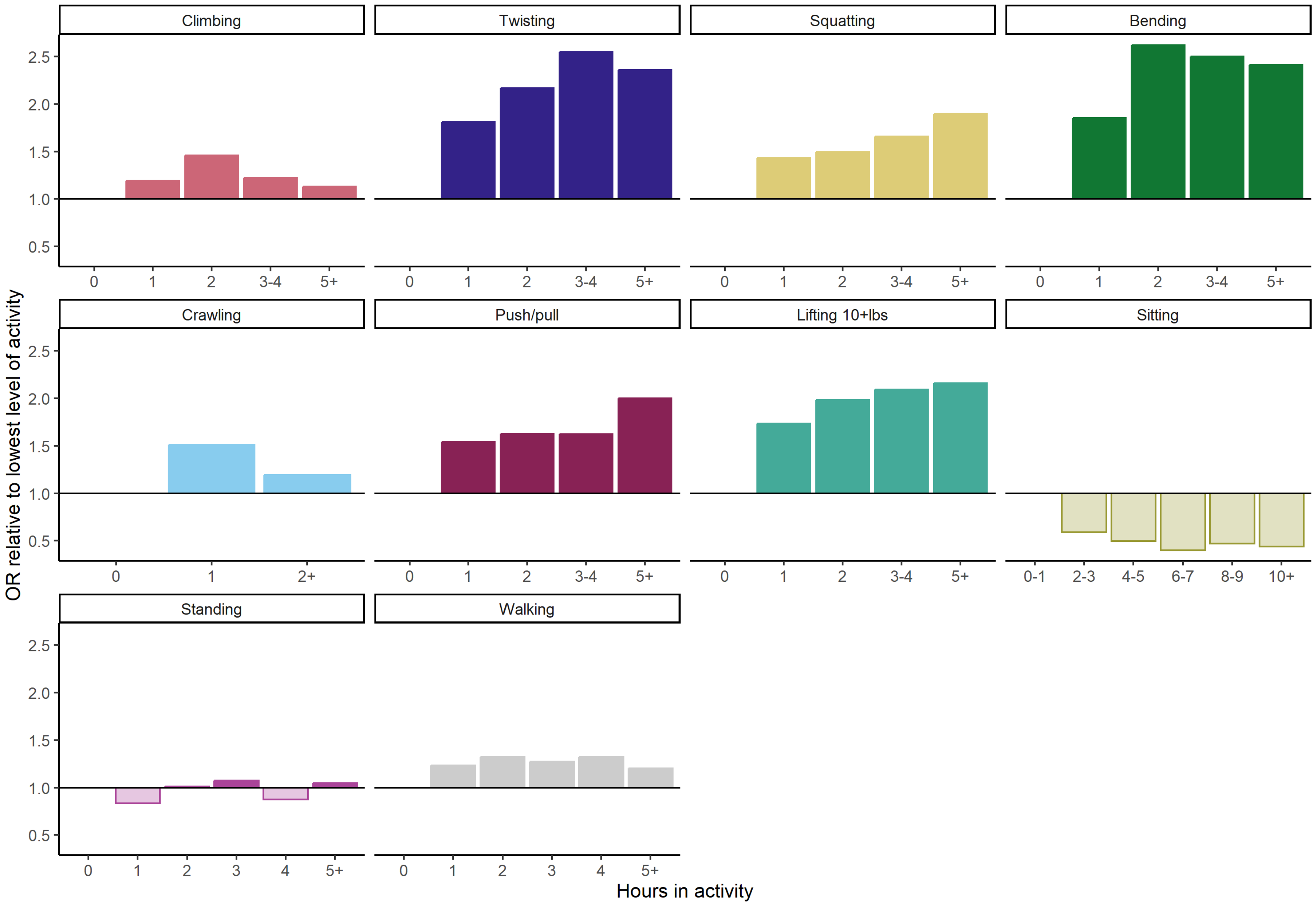

© 2025 Suri P et al. *JAMA Network Open.*

eTable 6. Associations between the number of times performing common physical activities during the past 24 hours and subsequent flares, imputed data[ab]

	Total		Non-flare periods		Flare periods		Unadjusted OR* (95% CI)		Adjusted OR[de] (95% CI)		p-value	Adjusted p-value[f]
**Climbing times (24 hours)**	7.81	(13.13)	8.16	(13.21)	6.97	(12.90)	1.03	(0.97, 1.09)	1.04	(0.97, 1.10)	0.262	0.262
**Twisting times (24 hours)**	7.67	(16.35)	7.29	(15.97)	8.58	(17.18)	1.17	(1.12, 1.22)	1.16	(1.11, 1.21)	<0.001	<0.001
**Squatting times (24 hours)**	7.04	(15.52)	6.99	(15.69)	7.15	(15.10)	1.12	(1.07, 1.17)	1.12	(1.07, 1.17)	<0.001	<0.001
**Bending times (24 hours)**	9.46	(16.72)	8.87	(15.97)	10.85	(18.27)	1.21	(1.16, 1.25)	1.20	(1.16, 1.26)	<0.001	<0.001
**Crawling times (24 hours)**	0.77	(4.46)	0.70	(4.34)	0.91	(4.73)	1.18	(1.04, 1.35)	1.17	(1.01, 1.35)	0.034	0.067
**Push/pull times (24 hours)**	5.30	(14.97)	4.99	(14.71)	6.04	(15.56)	1.10	(1.06, 1.15)	1.11	(1.06, 1.15)	<0.001	<0.001
**Lifting 10+lbs times (24 hours)**	12.32	(27.06)	12.04	(26.91)	12.98	(27.41)	1.07	(1.05, 1.10)	1.09	(1.06, 1.11)	<0.001	<0.001

[a]Of 9757 surveys completed by 416 participants, 9150 surveys were completed by 345 participants who were informative in the case-crossover analyses (i.e., the subset of participants who had ≥1 case period and ≥1 control period during 1-year follow-up).

[b]Sitting, standing, and walking are not included in this table because the metric of "number of times" is not relevant when considering engagement in these activities over a 24-hour period

[c]Mean (SD) hours spent in each activity during the past 24 hours (unadjusted). These values are presented for the whole sample of periods, for non-flare periods only, and for flare periods only.

[d]Odds ratio per 10 times increase in the number of times performed over the past 24 hours

[e]After adjustment for symptoms of depression,[27] post-traumatic stress disorder (PTSD),[28] general stress,[29,30] fear of movement (kinesiophobia),[31] catastrophizing,[32] and self-efficacy.[33]

[f]Holm-Bonferroni method

CI = Confidence interval; OR = odds ratio

© 2025 Suri P et al. *JAMA Network Open.*

eTable 7. Characteristics of participants completing vs. not completing 1-year RMDQ outcome (Aim 2)*

	Total (n=416)		Complete follow-up (n=317)		Incomplete follow-up (n=99)	
**Age, mean/SD**						
	47.54	10.86	47.52	10.49	47.61	12.10
**Birth sex**						
Male	306	75%	229	73%	77	80%
Female	104	25%	85	27%	19	20%
**Ethnicity (participant-reported)[a]**						
Hispanic or Latino	52	14%	39	13%	13	15%
**Race (participant-reported)[a]**						
American Indian or Alaska Native	4	1%	2	1%	2	2%
Asian	21	5%	14	5%	7	8%
Black or African-American	54	14%	40	13%	14	15%
Native Hawaiian or Other Pacific Islander	5	1%	3	1%	2	2%
White	279	70%	220	72%	59	63%
Multiracial	35	9%	26	9%	9	10%
**Marital status**						
Married/Living with S.O.	293	71%	227	72%	66	69%
Never married	37	9%	28	9%	9	9%
Separated/Divorced/Widowed	82	20%	61	19%	21	22%
**Body mass index, mean/SD**						
	30.37	5.69	30.28	5.58	30.64	6.04
**Education**						
Bachelor's degree or higher	193	47%	157	50%	36	38%
Less than bachelor's degree	219	53%	159	50%	60	62%
**Employment[c]**						
Working now	244	59%	190	60%	54	56%
Retired	93	23%	67	21%	26	27%
Disabled	79	19%	57	18%	22	23%
Other	47	11%	38	12%	9	9%
**Income**						
<$75,000	211	60%	162	59%	49	62%
$75,000+	141	40%	111	41%	30	38%
**Physical job demands**						
Not working	169	41%	127	40%	42	44%
<4	138	33%	110	35%	28	29%
4+	106	26%	80	25%	26	27%
**Job satisfaction**						
Not working	169	41%	127	40%	42	44%
Tertile 1: 5.0 to 10.0	102	25%	80	25%	22	23%
Tertile 2: >10.0 to 15.0	70	17%	58	18%	12	12%
Tertile 3: >15.0 to 35.0	72	17%	52	16%	20	21%
**Duration of low back pain**						
<3 months	14	3%	10	3%	4	4%
3 months to <1 year	15	4%	10	3%	5	5%
1 to 5 years	67	16%	49	16%	18	18%

© 2025 Suri P et al. *JAMA Network Open.*

eTable 7. Characteristics of participants completing vs. not completing 1-year RMDQ outcome (Aim 2)*

	Total (n=416)			Complete follow-up (n=317)		Incomplete follow-up (n=99)	
More than 5 years	318	77%		246	78%	72	73%
**Low back pain frequency**							
Less than half the days in the past 6 months	85	20%		73	23%	12	12%
At least half the days in the past 6 months	119	29%		91	29%	28	28%
Every day or nearly every day in the past 6 months	211	51%		152	48%	59	60%
**RMDQ, mean/SD**							
	12.19	5.81		11.51	5.75	14.38	5.47
**Low back pain NRS (Avg. 24hr), mean/SD**							
	4.46	2.21		4.29	2.14	5.02	2.34
**Cigarette smoking**							
Never smoked	230	55%		178	56%	52	53%
Current smoker	32	8%		25	8%	7	7%
Used to smoke, but have now quit	153	37%		114	36%	39	40%

SD=standard deviation, S.O.=significant other RMDQ=Roland Morris Disability Questionnaire, NRS=numeric rating scale, hr=hour

* Missing values for each variable are Birth sex: 6 (1%), Ethnicity: 36 (9%), Race: 18 (4%), Marital status: 4 (1%), Education: 4 (1%), Employment: 4 (1%), Income: 64 (15%), Physical job demands: 3 (1%), Job satisfaction: 3 (1%), Duration of low back pain: 2 (0%), Low back pain frequency: 1 (0%), Cigarette smoking: 1 (0%)

[a]Response options provided to participants included Hispanic or Latino, Not Hispanic or Latino, Unknown, and Not reported.

[b]Response options provided to participants included American Indian or Alaska Native, Asian, Black or African-American, Native Hawaiian or Other Pacific Islander, White, Unknown, and Not reported. If a participant selected multiple race categories, they were classified as Multiracial.

[c]Not mutually exclusive

© 2025 Suri P et al. *JAMA Network Open.*

eTable 8. Adjusted associations between 10 common physical activities performed during the first 8 weeks of study and 1-year functional outcomes as measured by the RMDQ, imputed data (n=416 participants)[a]

Variable	Est.	CI	p-value	Adjusted p-value[c]
**Climbing**[b]	0.02	(-0.26, 0.29)	0.912	1.000
**Twisting**[b]	-0.08	(-0.37, 0.21)	0.572	1.000
**Squatting**[b]	-0.20	(-0.59, 0.20)	0.320	1.000
**Bending**[b]	-0.03	(-0.34, 0.27)	0.825	1.000
**Crawling**[b]	0.09	(-0.68, 0.86)	0.814	1.000
**Push/pull**[b]	0.30	(-0.07, 0.67)	0.113	1.000
**Lifting 10+lbs**[b]	-0.04	(-0.27, 0.19)	0.741	1.000
**Sitting**[b]	-0.08	(-0.29, 0.13)	0.445	1.000
**Standing**[b]	-0.08	(-0.39, 0.23)	0.605	1.000
**Walking**[b]	-0.23	(-0.62, 0.16)	0.242	1.000

Est.= parameter estimate for the increase in 1-year RMDQ scores associated with an additional mean hour of activity at baseline (during the first 8 weeks of the study), CI = Confidence interval

[a]After adjustment for age, birth sex, body mass index, cigarette smoking, duration of low back pain,[9] low back pain frequency,[9] baseline Roland Morris Disability Questionnaire (RMDQ),[8] depression symptoms measured by the PROMIS Depression Short Form 8b,[17,21] post-traumatic stress disorder (PTSD) symptoms measured by the PTSD Checklist,[22] kinesiophobia measured by the 17-item TSK (Tampa Scale of Kinesiophobia),[23] catastrophizing measured by the CSQ-2 (Catastrophizing Strategies Scale, 2-item),[24] activity engagement and pain willingness as measured by the CPAQ-8 (Chronic Pain Acceptance Questionnaire, 8-item),[25] pain-related self-efficacy using the UW-PRSE-2 (UW Pain-Related Self-efficacy scale),[26] physical demands at work,[18] and job satisfaction.[19]

[b]Mean hours in activity in the past 24 hours, across all scheduled surveys during the first 8 weeks of the study

[c]Holm-Bonferroni method

© 2025 Suri P et al. *JAMA Network Open.*

eTable 9. Adjusted associations between 10 common physical activities performed during the first 8 weeks study and 1-year functional outcomes as measured by the RMDQ, complete case analysis[a]

Variable	Est.	CI	p-value	Adjusted p-value[c]
**Climbing**[b] (n=302)	0.04	(-0.22, 0.29)	0.779	1.000
**Twisting**[b] (n=302)	-0.13	(-0.44, 0.17)	0.399	1.000
**Squatting**[b] (n=302)	-0.22	(-0.57, 0.12)	0.207	1.000
**Bending**[b] (n=302)	-0.04	(-0.37, 0.30)	0.828	1.000
**Crawling**[b] (n=302)	0.06	(-0.75, 0.86)	0.889	1.000
**Push/pull**[b] (n=302)	0.08	(-0.42, 0.58)	0.757	1.000
**Lifting 10+lbs**[b] (n=302)	-0.16	(-0.40, 0.08)	0.189	1.000
**Sitting**[b] (n=301)	-0.14	(-0.34, 0.07)	0.189	1.000
**Standing**[b] (n=301)	-0.10	(-0.38, 0.18)	0.500	1.000
**Walking**[b] (n=300)	-0.34	(-0.66, -0.02)	0.036	0.360

Est.= parameter estimate for the increase in 1-year RMDQ scores associated with an additional mean hour of activity at baseline (during the first 8 weeks of the study), CI = Confidence interval

[a]After adjustment for age, birth sex, body mass index, cigarette smoking, duration of low back pain,[9] low back pain frequency,[9] baseline Roland Morris Disability Questionnaire (RMDQ),[8] depression symptoms measured by the PROMIS Depression Short Form 8b,[17,21] post-traumatic stress disorder (PTSD) symptoms measured by the PTSD Checklist,[22] kinesiophobia measured by the 17-item TSK (Tampa Scale of Kinesiophobia),[23] catastrophizing measured by the CSQ-2 (Catastrophizing Strategies Scale, 2-item),[24] activity engagement and pain willingness as measured by the CPAQ-8 (Chronic Pain Acceptance Questionnaire, 8-item),[25] pain-related self-efficacy using the UW-PRSE-2 (UW Pain-Related Self-efficacy scale),[26] physical demands at work,[18] and job satisfaction.[19]

[b]Mean hours in activity in the past 24 hours, across all scheduled surveys during the first 8 weeks of the study

[c]Holm-Bonferroni method

© 2025 Suri P et al. *JAMA Network Open.*

eTable 10. Adjusted associations between 10 common physical activities performed during the first 8 weeks of study and 1-year functional outcomes as measured by the RMDQ, among subset of participants informative in the Aim 1 case-crossover analyses, imputed data[a] (n=345)

Variable	Est.	CI	p-value	Adjusted p-value[c]
**Climbing**[b]	0.02	(-0.24, 0.28)	0.877	1.000
**Twisting**[b]	-0.11	(-0.43, 0.20)	0.475	1.000
**Squatting**[b]	-0.19	(-0.56, 0.17)	0.290	1.000
**Bending**[b]	-0.07	(-0.38, 0.25)	0.669	1.000
**Crawling**[b]	0.16	(-0.62, 0.93)	0.692	1.000
**Push/pull**[b]	0.12	(-0.36, 0.60)	0.616	1.000
**Lifting 10+lbs**[b]	-0.15	(-0.39, 0.09)	0.216	1.000
**Sitting**[b]	-0.07	(-0.28, 0.14)	0.509	1.000
**Standing**[b]	-0.07	(-0.35, 0.20)	0.604	1.000
**Walking**[b]	-0.26	(-0.61, 0.10)	0.154	1.000

Est.= parameter estimate for the increase in 1-year RMDQ scores associated with an additional mean hour of activity at baseline (during the first 8 weeks of the study), CI = Confidence interval
[a]After adjustment for age, birth sex, body mass index, cigarette smoking, duration of low back pain,[9] low back pain frequency,[9] baseline Roland Morris Disability Questionnaire (RMDQ),[8] depression symptoms measured by the PROMIS Depression Short Form 8b,[17,21] post-traumatic stress disorder (PTSD) symptoms measured by the PTSD Checklist,[22] kinesiophobia measured by the 17-item TSK (Tampa Scale of Kinesiophobia),[23] catastrophizing measured by the CSQ-2 (Catastrophizing Strategies Scale, 2-item),[24] activity engagement and pain willingness as measured by the CPAQ-8 (Chronic Pain Acceptance Questionnaire, 8-item),[25] pain-related self-efficacy using the UW-PRSE-2 (UW Pain-Related Self-efficacy scale),[26] physical demands at work,[18] and job satisfaction.[19]
[b]Mean hours in activity in the past 24 hours, across all scheduled surveys during the first 8 weeks of the study
[c]Holm-Bonferroni method

© 2025 Suri P et al. *JAMA Network Open.*

eTable 11 Non-linear associations between 10 common physical activities performed during the first 8 weeks of study and 1-year functional outcomes as measured by the RMDQ, imputed data (n=416 participants)[a]

Variable	Hours	Est.	CI	p-value[c]
**Lifting 10+lbs**[b]	0	-	(Ref.)	
	1	-0.74	(-2.23, 0.75)	0.328
	2+	-0.79	(-2.19, 0.61)	0.268
**Push/pull**[b]	0	-	(Ref.)	
	1	0.40	(-0.91, 1.70)	0.546
	2+	0.76	(-0.65, 2.17)	0.287
**Sitting**[b]	0-3	-	(Ref.)	
	4-5	0.18	(-1.43, 1.80)	0.824
	6-7	0.48	(-1.35, 2.31)	0.606
	8-9	-0.27	(-2.12, 1.58)	0.773
	10+	-0.80	(-2.94, 1.34)	0.462
**Standing**[b]	0-1	-	(Ref.)	
	2	-0.15	(-1.63, 1.33)	0.839
	3	0.22	(-1.50, 1.93)	0.801
	4	-0.36	(-2.24, 1.53)	0.708
	5+	-0.42	(-2.33, 1.49)	0.666
**Walking**[b]	0-1	-	(Ref.)	
	2	0.13	(-1.45, 1.71)	0.870
	3	-0.34	(-2.04, 1.35)	0.690
	4	-1.30	(-3.20, 0.59)	0.177
	5+	-1.11	(-3.21, 0.99)	0.297
**Bending**[b]	0	-	(Ref.)	
	1	-0.06	(-1.33, 1.20)	0.921
	2+	-0.29	(-1.52, 0.93)	0.638
**Climbing**[b]	0	-	(Ref.)	
	1	0.67	(-0.67, 2.01)	0.324
	2+	0.18	(-1.13, 1.49)	0.788
**Twisting**[b]	0	-	(Ref.)	
	1	-0.38	(-1.59, 0.83)	0.536
	2+	-0.84	(-2.11, 0.42)	0.190
**Squatting**[b]	0	-	(Ref.)	
	1	-0.56	(-1.84, 0.71)	0.383
	2+	-0.87	(-2.34, 0.61)	0.245
**Crawling**[b]	0	-	(Ref.)	
	1+	-1.76	(-3.41, -0.10)	0.038

Est.= parameter estimate for the increase in 1-year RMDQ scores associated with an additional mean hour of activity at baseline (during the first 8 weeks of the study), CI = Confidence interval

[a]After adjustment for age, birth sex, BMI, cigarette smoking, duration of low back pain,[9] low back pain frequency,[9] baseline RMDQ,[8] depression symptoms measured by the PROMIS Depression Short Form 8b,[17,21] post-traumatic stress disorder (PTSD) symptoms measured by the PTSD Checklist,[22] kinesiophobia measured by the 17-item TSK,[23] catastrophizing measured by the CSQ-2,[24] activity engagement and pain willingness as measured by the CPAQ-8,[25] pain-related self-efficacy using the UW-PRSE-2,[26] physical demands at work,[18] and job satisfaction[19]

[b]Mean hours in activity in the past 24 hours, across all scheduled surveys during the first 8 weeks of the study

[c]p-values not adjusted for multiple comparisons

© 2025 Suri P et al. *JAMA Network Open.*

eFigure 4. Graphical display of non-linear associations between 10 common physical activities performed during the first 8 weeks of study and 1-year functional outcomes as measured by the RMDQ, imputed data (n=416 participants)

© 2025 Suri P et al. *JAMA Network Open.*

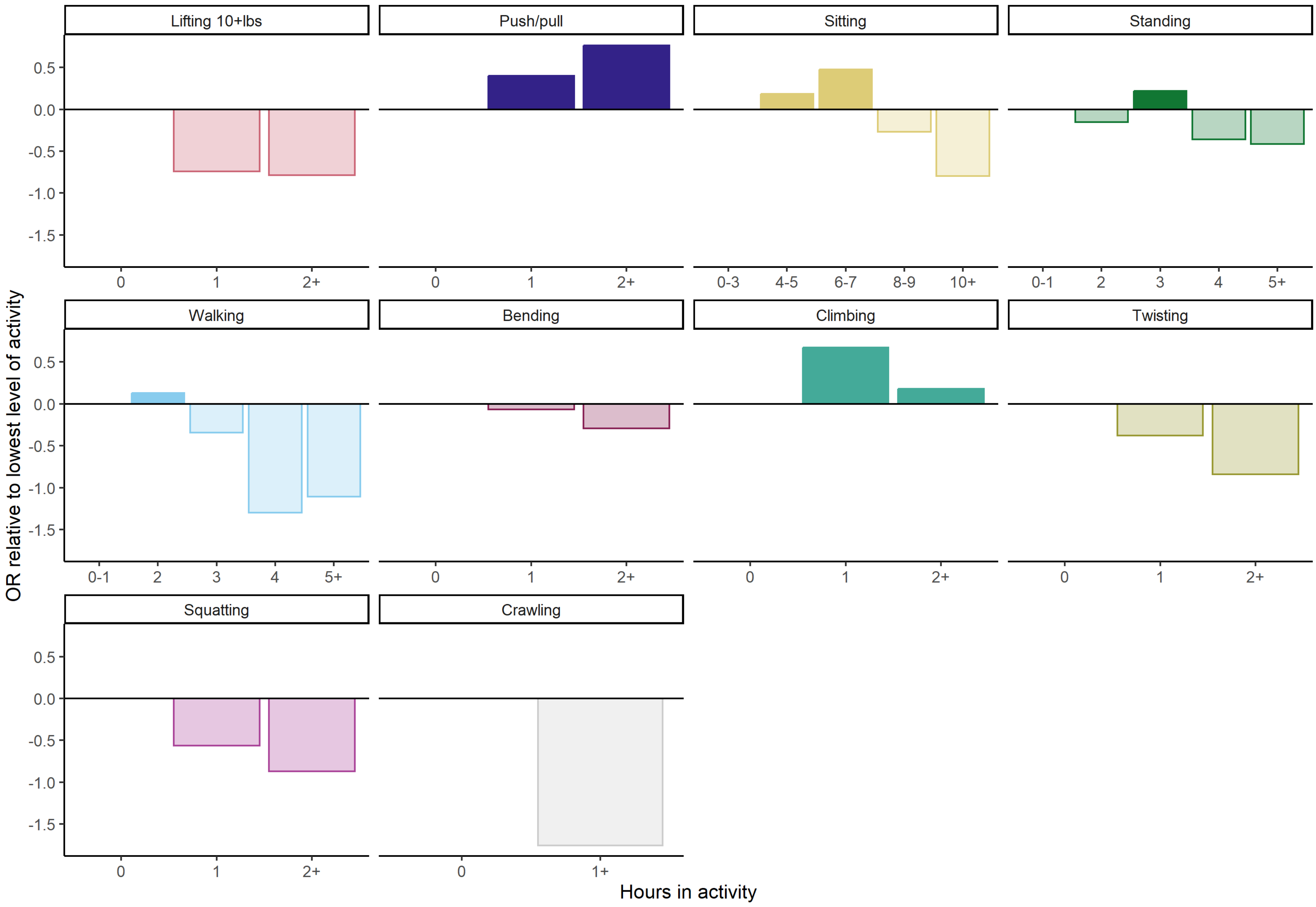

© 2025 Suri P et al. *JAMA Network Open.*

References

1. Suri P, Tanus AD, Torres N, et al. The Flares of Low back pain with Activity Research Study (FLAReS): study protocol for a case-crossover study nested within a cohort study. *BMC Musculoskelet Disord.* 2022;23(1):376.
2. Suri P, Tanus AD, Stanaway I, et al. Evaluating the representativeness of a cohort study of low back pain: Using electronic health record data to make direct comparisons of study participants with non-participants from the study population. *J Pain.* 2025;26:104730.
3. Maclure M, Mittleman MA. Should we use a case-crossover design? *Annu Rev Public Health.* 2000;21:193-221.
4. Suri P, Rainville J, de Schepper E, Martha J, Hartigan C, Hunter DJ. Do Physical Activities Trigger Flare-ups During an Acute Low Back Pain Episode?: A Longitudinal Case-Crossover Feasibility Study. *Spine (Phila Pa 1976).* 2018;43(6):427-433.
5. Gonge H, Jensen LD, Bonde JP. Do psychosocial strain and physical exertion predict onset of low-back pain among nursing aides? *Scand J Work Environ Health.* 2001;27(6):388-394.
6. Suri P, Korpak A, Timmons A, et al. Convergent validity of a person-dependent definition of a low back pain flare. *Pain.* 2025(In press).
7. Costa N, Ferreira ML, Setchell J, et al. A definition of 'flare' in low back pain (LBP): A multiphase process involving perspectives of individuals with LBP and expert consensus. *J Pain.* 2019;Epub ahead of print.
8. Roland M, Fairbank J. The Roland-Morris Disability Questionnaire and the Oswestry Disability Questionnaire. *Spine (Phila Pa 1976).* 2000;25(24):3115-3124.
9. Deyo RA, Dworkin SF, Amtmann D, et al. Report of the NIH Task Force on research standards for chronic low back pain. *J Pain.* 2014;15(6):569-585.
10. U.S. Department of Labor Office of Workers' Compensation Programs. Work Capacity Evaluation Musculoskeletal Conditions. https://www.dol.gov/sites/dolgov/files/owcp/dfec/regs/compliance/owcp-5c.pdf. Published 2014. Accessed 6/1/2024.
11. State of California Division of Workers' Compensation. Physician's Return-to-Work & Voucher Report. https://www.dir.ca.gov/dwc/forms/SJDB/10133.36.pdf. Published 2014. Accessed 3/12025, 2025.
12. Texas Department of Insurance Division of Worker's Compensation. Texas Workers' Compensation Work Status Report. 2019.
13. Industries WSDoLa. Activity Prescription Form. 2014.
14. Pope DP, Silman AJ, Cherry NM, Pritchard C, Macfarlane GJ. Validity of a self-completed questionnaire measuring the physical demands of work. *Scand J Work Environ Health.* 1998;24(5):376-385.
15. Stock SR, Fernandes R, Delisle A, Vezina N. Reproducibility and validity of workers' self-reports of physical work demands. *Scand J Work Environ Health.* 2005;31(6):409-437.
16. Wiktorin C, Karlqvist L, Winkel J. Validity of self-reported exposures to work postures and manual materials handling. Stockholm MUSIC I Study Group. *Scand J Work Environ Health.* 1993;19(3):208-214.
17. National Institutes of Health HEAL Initiative. National Institutes of Health HEAL Initiative: What are the core CDEs? Common Data Elements (CDEs) Program Web site. https://heal.nih.gov/data/common-data-elements. Published 2023. Accessed 6/7/2025.
18. Jakobsen MD, Sundstrup E, Persson R, Andersen CH, Andersen LL. Is Borg's perceived exertion scale a useful indicator of muscular and cardiovascular load in blue-collar workers with lifting tasks? A cross-sectional workplace study. *Eur J Appl Physiol.* 2014;114(2):425-434.
19. Rentsch JR, Steeel RP. Construct and Concurrent Validation of the Andrews and Withey Job Satisfaction Questionnaire. *Educational and Psychological Measurement.* 1992;52:357-367.
20. Farrar JT, Young JP, Jr., LaMoreaux L, Werth JL, Poole RM. Clinical importance of changes in chronic pain intensity measured on an 11-point numerical pain rating scale. *Pain.* 2001;94(2):149-158.
21. Pilkonis PA, Choi SW, Reise SP, et al. Item banks for measuring emotional distress from the Patient-Reported Outcomes Measurement Information System (PROMIS(R)): depression, anxiety, and anger. *Assessment.* 2011;18(3):263-283.

© 2025 Suri P et al. *JAMA Network Open.*

22. Magruder K, Yeager D, Goldberg J, et al. Diagnostic performance of the PTSD checklist and the Vietnam Era Twin Registry PTSD scale. *Epidemiol Psychiatr Sci.* 2015;24(5):415-422.
23. Roelofs J, Goubert L, Peters ML, Vlaeyen JW, Crombez G. The Tampa Scale for Kinesiophobia: further examination of psychometric properties in patients with chronic low back pain and fibromyalgia. *Eur J Pain.* 2004;8(5):495-502.
24. Tan G, Nguyen Q, Cardin SA, Jensen M. Validating the use of two-item measures of pain beliefs and coping strategies for a veteran population. *Journal of Pain.* 2006;7:252-260.
25. Fish RA, McGuire B, Hogan M, Morrison TG, Stewart I. Validation of the chronic pain acceptance questionnaire (CPAQ) in an Internet sample and development and preliminary validation of the CPAQ-8. *Pain.* 2010;149(3):435-443.
26. Amtmann D, Bamer AM, Liljenquist KS, et al. The Concerns About Pain (CAP) Scale: A Patient-Reported Outcome Measure of Pain Catastrophizing. *J Pain.* 2020;21(11-12):1198-1211.
27. Zimmerman M, Ruggero CJ, Chelminski I, et al. Developing brief scales for use in clinical practice: the reliability and validity of single-item self-report measures of depression symptom severity, psychosocial impairment due to depression, and quality of life. *J Clin Psychiatry.* 2006;67(10):1536-1541.
28. Tiet QQ, Schutte KK, Leyva YE. Diagnostic accuracy of brief PTSD screening instruments in military veterans. *J Subst Abuse Treat.* 2013;45(1):134-142.
29. Arapovic-Johansson B, Wahlin C, Kwak L, Bjorklund C, Jensen I. Work-related stress assessed by a text message single-item stress question. *Occup Med (Lond).* 2017;67(8):601-608.
30. Elo AL, Leppanen A, Jahkola A. Validity of a single-item measure of stress symptoms. *Scand J Work Environ Health.* 2003;29(6):444-451.
31. Vangronsveld KL, Peters M, Goossens M, Vlaeyen J. The influence of fear of movement and pain catastrophizing on daily pain and disability in individuals with acute whiplash injury: a daily diary study. *Pain.* 2008;139(2):449-457.
32. Jensen MP, Keefe FJ, Lefebvre JC, Romano JM, Turner JA. One- and two-item measures of pain beliefs and coping strategies. *Pain.* 2003;104(3):453-469.
33. Amtmann D, Bamer AM, Cook KF, Askew RL, Noonan VK, Brockway JA. University of Washington self-efficacy scale: a new self-efficacy scale for people with disabilities. *Arch Phys Med Rehabil.* 2012;93(10):1757-1765.
34. Zhang Y, Zhang B, Wise B, Niu J, Zhu Y. Statistical approaches to evaluating the effect of risk factors on the pain of knee osteoarthritis in longitudinal studies. *Curr Opin Rheumatol.* 2009;21(5):513-519.
35. Rubin DB. *Multiple Imputation for Nonresponse in Surveys.* New York: John Wiley and Sons; 1987.
36. van Buuren S, Groothuis-Oudshoorn K. MICE: Multivariate Imputation by Chained Equations in R. *Journal of Statistical Software.* 2011;45(3):1-67.
37. Perkins NJ, Cole SR, Harel O, et al. Principled Approaches to Missing Data in Epidemiologic Studies. *Am J Epidemiol.* 2018;187(3):568-575.
38. Moons KG, Donders RA, Stijnen T, Harrell FE, Jr. Using the outcome for imputation of missing predictor values was preferred. *J Clin Epidemiol.* 2006;59(10):1092-1101.
39. Huque MH, Carlin JB, Simpson JA, Lee KJ. A comparison of multiple imputation methods for missing data in longitudinal studies. *BMC Med Res Methodol.* 2018;18(1):168.
40. Robitzch A, Grund S. miceadds: Some Additional Multiple Imputation Functions, Especially for 'mice. R package version 3.17-44. . https://cran.r-project.org/web/packages/miceadds/index.html. Published 2024. Accessed.

© 2025 Suri P et al. *JAMA Network Open.*
