# Supplementary material for: Transient and Long-Term Risks of Common Physical Activities in People With Low Back Pain
Source: JAMA Netw Open. 2025 Dec 9;8(12):e2547915. doi: 10.1001/jamanetworkopen.2025.47915 (PMC12690428; doi:10.1001/jamanetworkopen.2025.47915)
Supplement: Supplement 2. — Group Information [file jamanetwopen-e2547915-s002.pdf]

Supplemental Online Content: Nonauthor Collaborators
*First name, last name, and suffix (if applicable) are required and will appear in PubMed.

*Group Name(s): FLAReS Collaborators							
***First Name and Middle Initial(s)**	***Last Name**	***Suffix (eg, Jr, III)**	Academic Degrees	Institution	Location (city, state/province, country)	Role or Contribution, eg, chair, principal investigator	Group (if more than 1 Group listed in the byline) and/or Subgroup (eg, Steering Committee)
Srecharan	Gorukanti		MD	Advocate Aurora Health	Oak Lawn, IL	Acquisition of data	NA
Albert H.	Chang		MD	Virginia Mason Franciscan Health	Seattle, IL	Acquisition of data	NA
Bianca	Dowling		MPH	University of South Florida	Tampa, FL	Acquisition of data	NA
Jihong	Min		MD	Vanderbilt University	Nashville, TN	Acquisition of data	NA
Jason S.	Silver		MD, PhD	University of Washington	Seattle, WA	Acquisition of data	NA
