# Supplementary material for: Transient and Long-Term Risks of Common Physical Activities in People With Low Back Pain
Source: JAMA Netw Open. 2025 Dec 9;8(12):e2547915. doi: 10.1001/jamanetworkopen.2025.47915 (PMC12690428; doi:10.1001/jamanetworkopen.2025.47915)
Supplement: Supplement 3. — Data Sharing Statement [file jamanetwopen-e2547915-s003.pdf]

# Data Sharing Statement

Suri. Transient and Long-Term Risks of Common Physical Activities in People With Low Back Pain. *JAMA Netw Open*. Published December 09, 2025.
doi:10.1001/jamanetworkopen.2025.47915

**Data**
**Data available:** No

**Additional Information**
**Explanation for why data not available:** The study's informed consent processes do not allow study datasets containing individual-level data to be made available outside of the federal government.
